# Single-cell analysis of the fate of c-kit-positive bone marrow cells

**DOI:** 10.1038/s41536-017-0032-1

**Published:** 2017-10-16

**Authors:** Anna Czarna, Fumihiro Sanada, Alex Matsuda, Junghyun Kim, Sergio Signore, João D. Pereira, Andrea Sorrentino, Ramaswamy Kannappan, Antonio Cannatà, Toru Hosoda, Marcello Rota, Filippo Crea, Piero Anversa, Annarosa Leri

**Affiliations:** 1 Departments of Anesthesia and Medicine, and Division of Cardiovascular Medicine, Brigham and Women’s Hospital, Harvard Medical School, Boston, MA 02115 USA; 2 Cardiocentro Ticino, University of Zurich, Lugano, 6900 Switzerland; 3 Tokai University Institute of Innovative Science and Technology, Isehara, Kanagawa 259-1193 Japan; 4 Department of Physiology, New York Medical College, Valhalla, NY 10595 USA; 5 Department of Cardiovascular Sciences, Catholic University of the Sacred Heart, Agostino Gemelli Polyclinic, Rome, 00168 Italy

## Abstract

The plasticity of c-kit-positive bone marrow cells (c-kit-BMCs) in tissues different from their organ of origin remains unclear. We tested the hypothesis that c-kit-BMCs are functionally heterogeneous and only a subgroup of these cells possesses cardiomyogenic potential. Population-based assays fall short of identifying the properties of individual stem cells, imposing on us the introduction of single cell-based approaches to track the fate of c-kit-BMCs in the injured heart; they included viral gene-tagging, multicolor clonal-marking and transcriptional profiling. Based on these strategies, we report that single mouse c-kit-BMCs expand clonally within the infarcted myocardium and differentiate into specialized cardiac cells. Newly-formed cardiomyocytes, endothelial cells, fibroblasts and c-kit-BMCs showed in their genome common sites of viral integration, providing strong evidence in favor of the plasticity of a subset of BMCs expressing the c-kit receptor. Similarly, individual c-kit-BMCs, which were infected with multicolor reporters and injected in infarcted hearts, formed cardiomyocytes and vascular cells organized in clusters of similarly colored cells. The uniform distribution of fluorescent proteins in groups of specialized cells documented the polyclonal nature of myocardial regeneration. The transcriptional profile of myogenic c-kit-BMCs and whole c-kit-BMCs was defined by RNA sequencing. Genes relevant for engraftment, survival, migration, and differentiation were enriched in myogenic c-kit-BMCs, a cell subtype which could not be assigned to a specific hematopoietic lineage. Collectively, our findings demonstrate that the bone marrow comprises a category of cardiomyogenic, vasculogenic and/or fibrogenic c-kit-positive cells and a category of c-kit-positive cells that retains an undifferentiated state within the damaged heart.

## Cardiovascular biology: Variation among heart-forming bone marrow cells

A select group of bone marrow cells (BMCs) with the capacity to regenerate the heart are not all the same. Working with mouse cells, a team led by Annarosa Leri used single cell-based analytical techniques to test whether all BMCs that express a cell surface marker called c-kit possess the ability to form new heart tissue. They found that these BMCs, despite their shared expression of c-kit, were not a uniform population. Only a subset could give rise to various cell lineages in the heart. Others remained in an undifferentiated state and retained their bone marrow identity, even within the damaged heart. The findings could help explain why researchers have reported such disparate results in the past when assessing the heart repairing potential of c-kit-positive BMCs.

## Introduction

Following our original publication in 2001 reporting the ability of c-kit-positive bone marrow cells (c-kit-BMCs) to regenerate cardiomyocytes and coronary vessels in the infarcted mouse heart,^1^ several studies have evaluated the role of BMCs in cardiac repair. However, both experimentally and clinically, this research has focused mostly on cell populations different from c-kit-BMCs; they included bone marrow mononuclear cells (BM-MNCs), endothelial progenitor cells, mesenchymal stem cells, purified CD34-positive-BMCs, SSEA1-positive-BMCs, CD133-positive-BMCs and very small embryonic-like-BMCs.^2^ The use of distinct pools of BMCs has made the comparison among studies rather complex.^3,4^ Despite this limitation, agreement has been reached in regard to the mechanisms of action of these multiple BMC classes. It is well-accepted that the majority of BMCs acts as a reservoir of cytokines and growth factors, which influence in a paracrine fashion endogenous cardiac stem cells (CSCs), cardiomyocytes and vascular cells.^2^ Additionally, BMCs have shown various degrees of vasculogenic potential having little or no ability to form cardiomyocytes.^2,3^

The fate of the subset of BMCs expressing c-kit in the injured heart and their potential role in myocardial regeneration remains controversial. De novo cardiomyogenesis has been attributed to transdifferentiation of c-kit-BMCs, growth activation of recipient progenitors or fusion of the delivered cells with pre-existing cardiomyocytes.^5^ Moreover, it has been suggested that c-kit-BMCs fail to adopt a cardiac phenotype and retain their hematopoietic identity.^6^ Understanding the basis of these conflicting results is important for the recognition of the function that c-kit-BMCs may have clinically. Differences in experimental outcome may be attributed to the use of cells that share the expression of the c-kit receptor but are otherwise phenotypically distinct. Lineage negative and lineage positive c-kit-BMCs, c-kit^+^-Thy1.1^lo^-Lin^-^-Sca-1^+^ BMCs, estrogen receptor α-positive c-kit-BMCs and c-kit-positive-Nkx2.5-positive BMCs have been tested and contrasting findings have been published.^6–8^ To avoid pre-selection for additional antigens, we have elected to study the entire compartment of BMCs expressing the receptor tyrosine kinase c-kit. This approach allowed us to define the functional heterogeneity of c-kit-BMCs, which was determined at the single-cell level by employing intracellular tags unique to individual c-kit-BMCs and their progeny.

The clonal fate of single c-kit-BMCs in vivo was established first by lentiviral gene-tagging, a powerful and accurate methodology for the identification of the descendants formed by lineage specification of individual stem cells.^9^ Thus far, this approach has been applied to the analysis of hematopoiesis, neurogenesis and retinal regeneration,^10–13^ but has not been utilized to characterize the function of c-kit-BMCs in the development of non-hematopoietic tissues and the myocardium in particular. This analysis was then expanded to the recognition of the molecular signature of single-cell-derived clonal populations of c-kit-BMCs capable of generating cardiomyocytes in vivo.

Viral gene tagging and RNA sequencing require dissociation of the tissue preventing the visualization of the morphological aspects of cardiac repair. Previous work from our laboratory has documented by immunolabeling and confocal microscopy the characteristics of the regenerated myocardium following the delivery of GFP-labeled c-kit-BMCs. In the current study, we added a new level of complexity to this strategy by introducing multicolor cell tagging;^14^ lentiviral vectors carrying distinct fluorescent proteins give rise to a large spectrum of color gradations in the infected cells and their daughter cells. The variety of different nuances generated by the mixture of the primary colors (red, green, blue) allowed the recognition of the polyclonal nature of myocardial reconstitution. Collectively, our findings demonstrate that the bone marrow comprises a category of cardiomyogenic, vasculogenic and/or fibrogenic c-kit-positive cells and a category that retains an undifferentiated state within the damaged heart.

## Results

### Viral gene tagging and phenotype of c-kit-BMCs

Mouse c-kit-BMCs were enriched with immunomagnetic beads and cultured in non-coated dishes for 2 days in the presence of growth factors to increase the fraction of cycling cells and their sensitivity to lentiviral infection. Floating cells were transferred to RetroNectin-coated dishes and cultured for an additional 3 days in the presence of viral particles carrying GFP to obtain fluorescently-labeled cells. To determine whether c-kit-BMCs form a cardiomyocyte progeny in vivo, myocardial infarction was induced by coronary ligation in syngeneic mice (*n* = 8). Shortly after coronary occlusion, 1 × 10^5^ FACS-sorted GFP-positive c-kit-BMCs were injected in four different sites of the region bordering the infarct. All animals were treated with GFP-positive c-kit-BMCs collected from the same preparation. Two weeks after surgery, the treated-infarcted hearts were enzymatically dissociated with collagenase to obtain a single cell suspension.

**Fig. 1:**
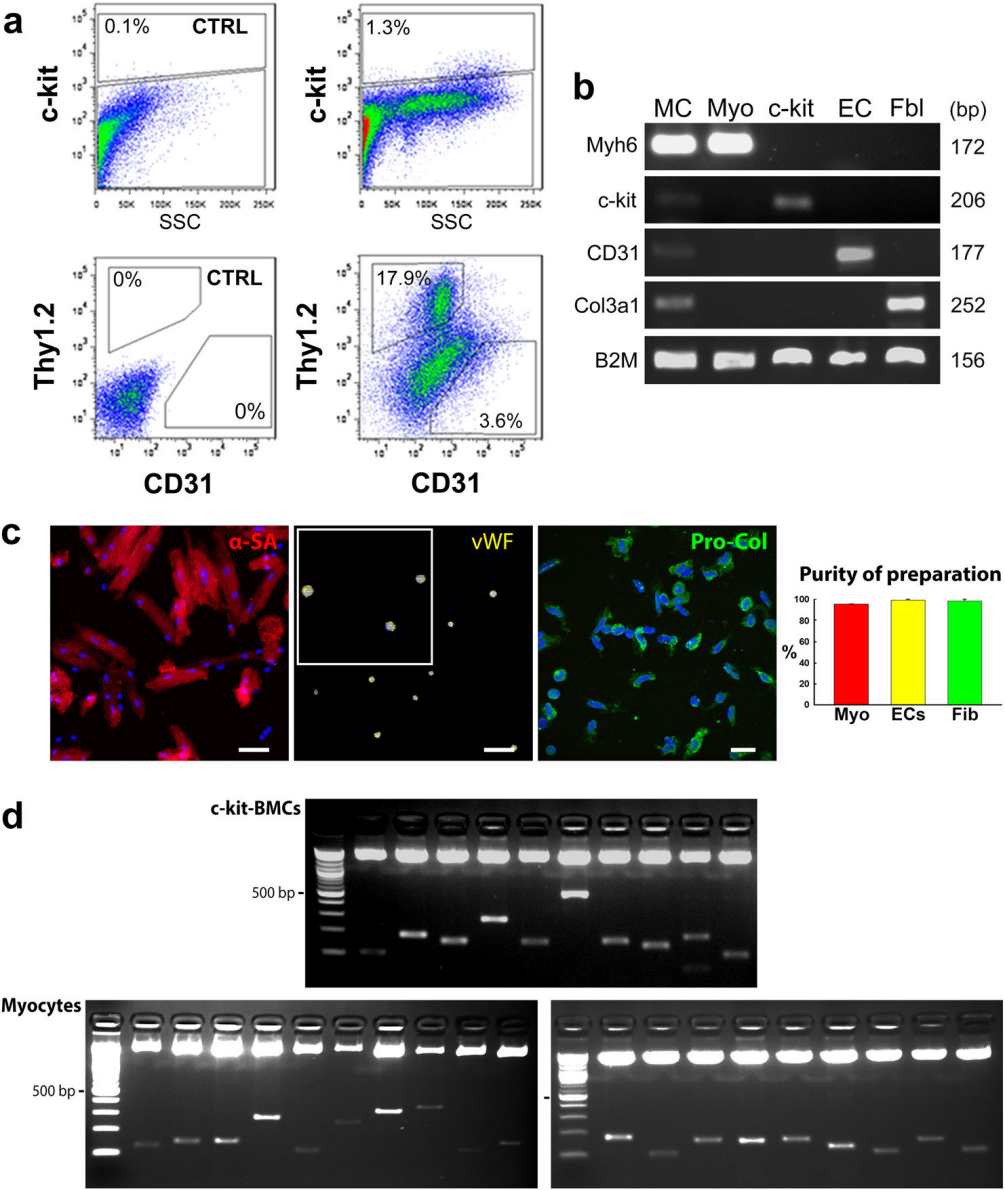
c-kit-BMCs acquire distinct cardiac cell phenotypes in vivo. **a** Representative scatter plots illustrating the expression of c-kit, Thy1.2 and CD31 in cardiac cell populations isolated from c-kit-BMC-treated infarcted hearts. The percentage of positive cells is indicated. CTRL: isotype control; SSC: side scatter. **b** Transcripts for α-myosin heavy chain (*Myh6*), c-kit, CD31, collagen type III α-1 (*Col3a1*) and β-2 microglobulin (*B2M*) in isolated cardiomyocytes (Myo), c-kit-BMCs (c-kit), endothelial cells (ECs) and fibroblasts (Fbl). Myocardium (first lane, MC) was used as control. bp: base pairs. **c** Isolated cardiomyocytes expressing α-sarcomeric actin (α-SA, red), ECs expressing von Willebrand factor (vWF, yellow) and fibroblasts expressing procollagen (Pro-Col, green) are shown. Quantitative data are presented as mean ± SD. Scale bars: Left and central panels = 50 µm; Right panel = 20 µm. **d** PCR products run on agarose gel correspond to the sites of integration of the viral genome in the DNA of c-kit-BMCs and myocytes. These images correspond to representative examples of experiments conducted in 8 mice. The upper band shows the pCR4-TOPO TA vector. Molecular mass: 100 bp incremental ladders

Myocytes were purified by differential centrifugation, while endothelial cells (ECs), fibroblasts and c-kit-positive cells were sorted by flow-cytometry based on the expression of CD31, Thy1.2, and c-kit. ECs were positive for CD31, and negative for c-kit and Thy1.2, fibroblasts were positive for Thy1.2 (refs. 15, 16) and negative for c-kit and CD31, and c-kit-positive cells expressed this epitope but were negative for CD31 and Thy1.2 (Fig. 1a). RT-PCR was employed to determine the purity of each cell preparation; transcripts for α-myosin heavy chain (*Myh6*), CD31, and procollagen (*Col3a1*) were restricted, respectively, to myocytes, ECs, and fibroblasts (Fig. 1b). The expression of c-kit in these three differentiated cell populations was evaluated to assess the presence of contaminant c-kit-positive cells; c-kit mRNA was not found in myocyte, EC and fibroblast preparations. Additionally, aliquots from each cell sample were fixed in paraformaldehyde and their purity was determined by immunolabeling and confocal microscopy (Fig. 1c). In all cases, the level of contamination from other cardiac cells was negligible, indicating that our protocol of cell type separation was satisfactory for the analysis of the site of viral integration in the genome of each cardiac cell population. Vascular smooth muscle cells were not included in this analysis; they represent a minimal fraction of the cardiac cell populations and cannot be acquired in reasonable quantity. Importantly, tissue digestion may be associated with loss of myocardial cells changing the proportion of the different cell compartments present in vivo. Because of this potential variable, data could not be analyzed in a quantitative form.

### Sites of viral integration in c-kit-BMCs, cardiomyocytes, ECs and fibroblasts

The viral integration site in the DNA of the mother cell is inherited by the daughter cells, constituting a unique clonal tag that unmasks the parental relationship between phenotypically distinct cell types. The insertion site of the GFP gene corresponds to a specific DNA sequence flanking the viral genome, and this genomic region was identified by PCR. DNA was extracted from myocytes, ECs, fibroblasts, and c-kit-positive BMCs isolated, as discussed above, from cell-treated infarcted hearts. PCR products were run on agarose gel generating multiple bands of distinct molecular mass (Fig. 1d).

By sequence analysis, the purified DNA contained the viral and mouse genome, and, thereby, corresponded to viral integrant sites (Supplementary Fig. S1). A total of 111 insertion sites were identified in 7 out of 8 independent experiments; 65 reflected different sites of integration (Supplementary Fig. S2a). Of the 65 proviral integrants, 13 were restricted to cardiomyocytes indicating retrospectively that these cells derived from myogenic mother c-kit-BMCs; 18 were restricted to ECs which derived from vasculogenic mother c-kit-BMCs; and 10 were restricted to fibroblasts which derived from fibrogenic mother c-kit-BMCs. The 12 cases in which the site of integration was restricted to c-kit-BMCs only were interpreted as self-renewing cells which did not acquire cardiovascular phenotypes, possibly retaining their hematopoietic identity. In 12 cases, common viral integration sites were detected in c-kit-BMCs, myocytes, ECs, and fibroblasts in various combinations, strengthening the presence of a multi-lineage liaison between the delivered c-kit-BMCs and the various cardiac cell types (Supplementary Fig. S2b). Thus, clonal expansion and commitment of individual c-kit-BMCs occur in vivo, supporting the view that these cells regenerate the infarcted heart.

### Cardiomyogenic fate of clonal c-kit-BMCs in vivo

**Fig. 2:**
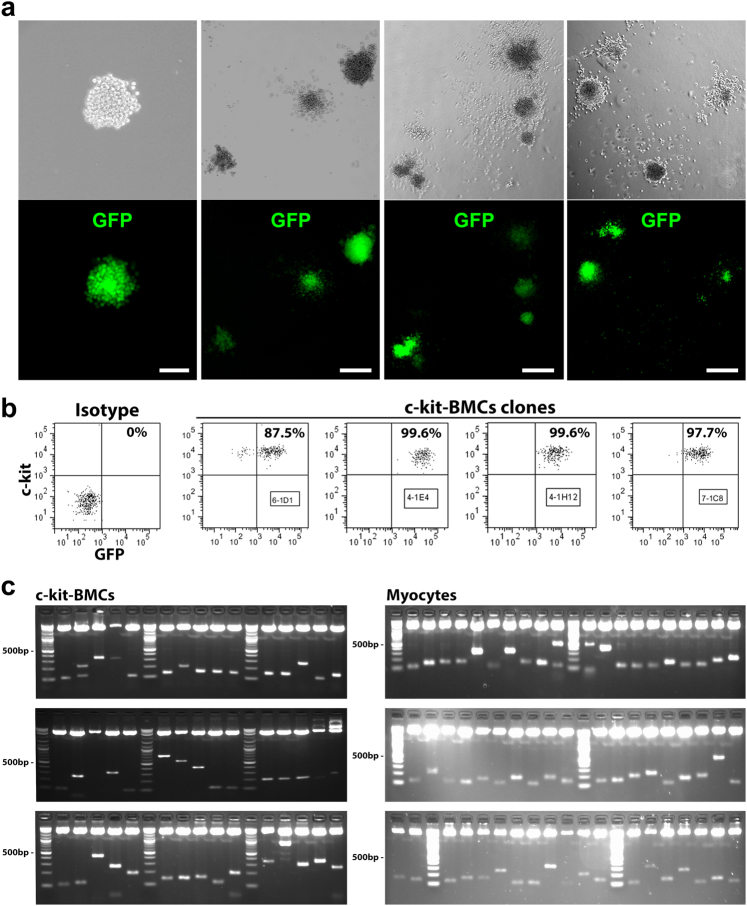
Myogenic and non-myogenic clonal c-kit-BMCs. **a** Sorted GFP-positive-c-kit-BMCs, plated at limiting dilution in semi-solid medium, generate single cell-derived clones (upper panels, phase contrast micrographs; lower panels, native GFP fluorescence). Scale bars: first panel = 50 µm; second panel = 100 µm; third and fourth panels = 200 µm. **b** Scatter plots of c-kit and GFP expression in clonal c-kit-BMCs. The numbers in the boxes correspond to the sampled cell clones. **c** Three weeks after myocardial infarction and injection of clonal GFP-positive-c-kit-BMCs, sites of viral integrations were detected in aliquots of the delivered cells and in isolated regenerated cardiomyocytes. The PCR products correspond to the sites of integration of the viral genome in the DNA of c-kit-BMCs and cardiomyocytes. Molecular mass: 100 bp incremental ladders

Our observations raised the possibility that phenotypically distinct populations of c-kit-BMCs have a different capacity to form cardiomyocytes. To test this hypothesis with a molecular strategy independent from immunolabeling and confocal microscopy, immuno-sorted c-kit-BMCs were infected with a GFP-lentivirus. Subsequently, cells were FACS-sorted for c-kit and GFP, and single cells were deposited at limiting dilution in semi-solid medium for clonal growth.^17^ The percentage of c-kit-positive cells in the clones examined by FACS varied from 87.5% to nearly 100% (Fig. 2a, b). Fifteen clones were utilized to obtain three distinct cell preparations, each consisting of a mixture of 5 clones; 1 × 10^5^ cells were injected in the border zone of acutely infarcted hearts and mice were sacrificed 21 days later. Three groups of infarcted mice (group 1: *n* = 7; group 2: *n* = 6; group 3: *n* = 8) were included in this analysis.

**Fig. 3:**
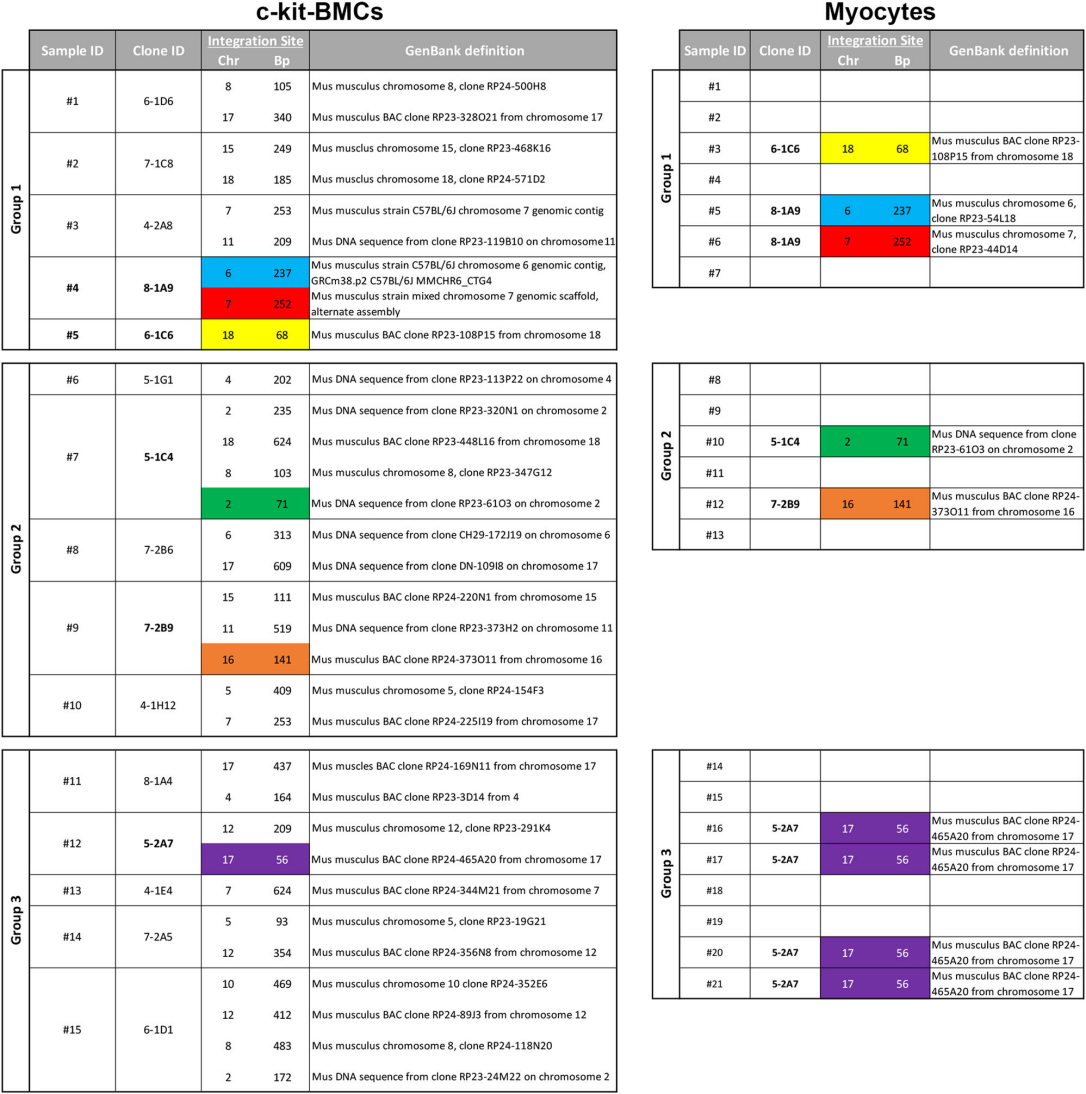
Detection of integration sites in c-kit-BMCs and cardiomyocytes. Results obtained in three groups of infarcted mice treated with clonal GFP-positive c-kit-BMCs are illustrated. Viral insertion sites were identified by PCR and sequencing in c-kit-BMCs (left column) and cardiomyocytes (right colum). Identical integration sites in the two cell types are represented by the same color. Molecular mass: 100 bp incremental ladders

Following enzymatic digestion and cardiomyocyte isolation from 21 hearts, the site of viral integration in the cardiomyocyte DNA was determined and compared with that present in aliquots of clonal c-kit-BMC preparations, preserved prior to transplantation in vivo (Fig. 2c). When the same viral insertion site was found in the two cell classes, i.e., c-kit-BMCs and dissociated cardiomyocytes, the injected clones were defined as myogenic (Fig. 3). Clones lacking this association were defined as non-myogenic: of the 15 clones, 5 were myogenic and 10 were non-myogenic. A common site of integration was found between cardiomyocytes and two of the c-kit-BMC clones in the first group of injected mice, two of the c-kit-BMC clones delivered to the second group of mice and one of the c-kit-BMC clones transplanted in the third group of mice (Fig. 3, color-coded). An inevitable limitation of this assay is that clonal expansion in vitro may affect partly the developmental choice of the expanded cells.

### Gene expression profile of myogenic c-kit-BMCs

**Fig. 4:**
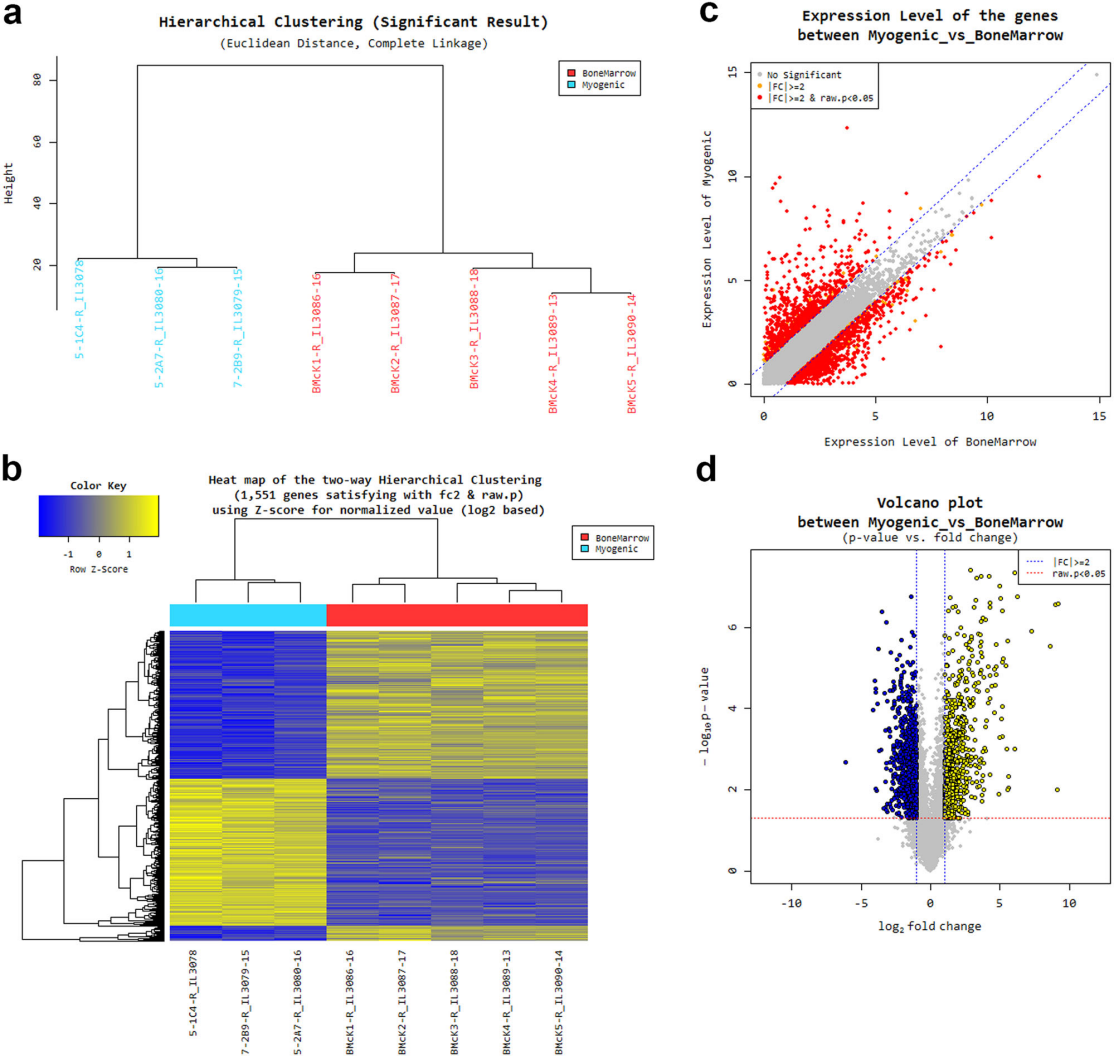
RNA sequencing of myogenic clonal c-kit-BMCs and whole c-kit-BMCs. **a** Hierarchial clustering analysis of differentially expressed genes (DEGs) in myogenic clonal c-kit-BMCs (myogenic, blue) and whole c-kit-BMCs (Bone marrow, red). **b** Heatmap of the two-way hierarchical clustering (see panel a) representing graphically the similarity of gene expression patterns between samples. **c** Scatter plot representing gene expression levels in the two cell groups. The statistical significance (*P* < 0.05) of the fold change (FC) difference is indicated. **d** Volcano plot representing gene expression levels in the two cell groups

The molecular signature of myogenic clonal c-kit-BMCs (*n* = 3) and whole c-kit-BMCs (*n* = 5) was determined by RNA sequencing. The entire population of c-kit-BMCs represents the adequate comparative sample for the detection of specific identifiers of the myogenic subset. This approach allows the prospective isolation of a cell pool characterized by high propensity to regenerate the damaged heart. Known and novel transcripts, and novel alternative splicing variants of known transcripts were assembled with Cufflinks, and the abundance of the normalized value of transcripts was determined. By the whole transcriptome sequencing, 1551 differentially expressed genes (DEGs), which showed a statistically significant (*P* < 0.05) fold-change difference ≥ 2, were found (Fig. 4; Supplementary Dataset S1); in myogenic c-kit-BMCs, 735 and 816 genes were upregulated and downregulated, respectively.

**Fig. 5:**
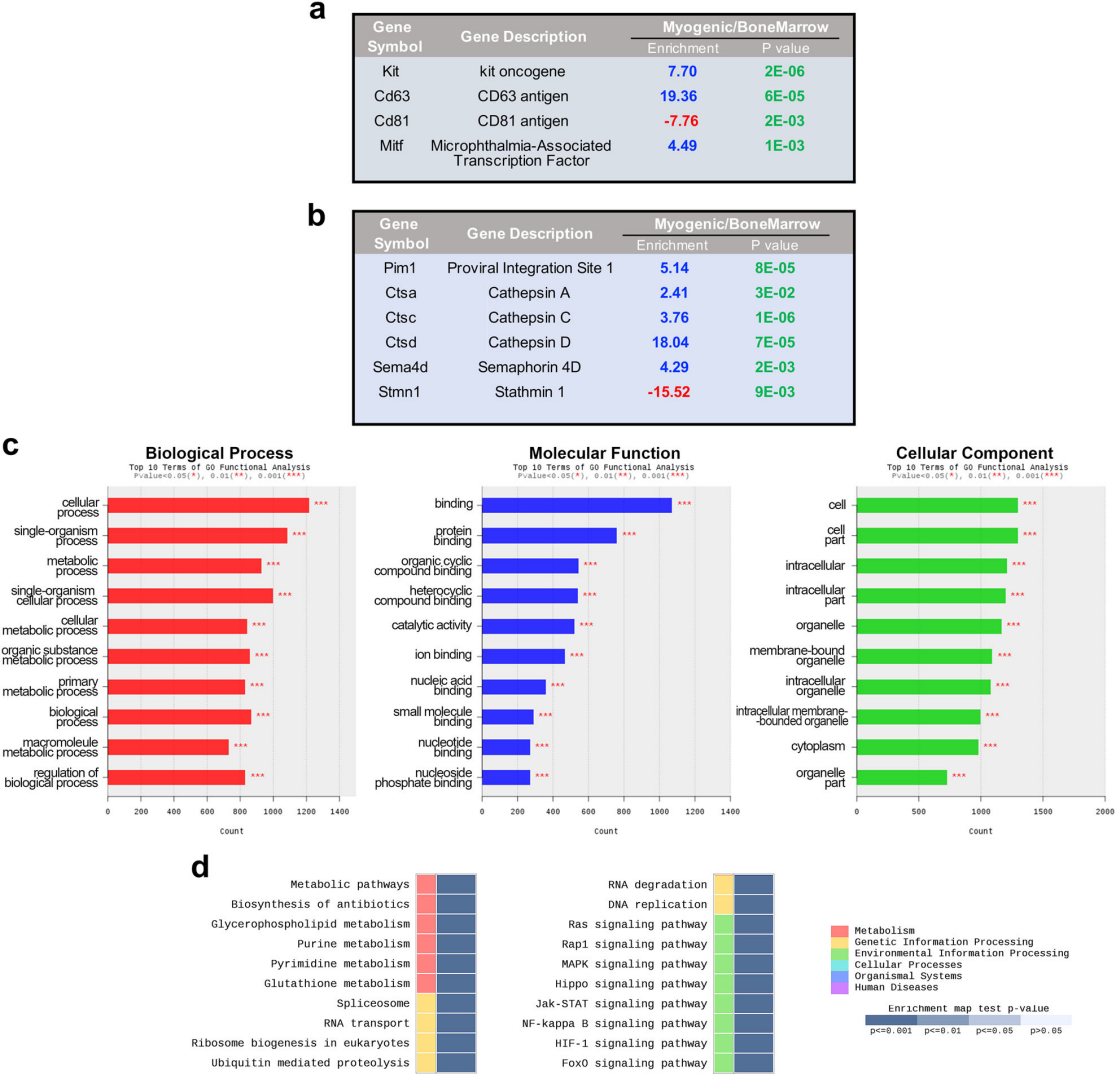
Gene expression profile of myogenic c-kit-BMCs. **a**, **b** Selected DEGs (see text for detail) in myogenic c-kit-BMCs and whole c-kit-BMCs. **c** Top 10 terms of GO functional analysis for the three categories: biological process, molecular function and cellular component. **d** Enrichment map (KEGG database) of annotated gene sets in myogenic c-kit-BMCs vs. whole bone marrow. The color gradient shows the range of P-values; the top 20 gene sets are included (for the complete map, see Supplementary Fig. S3)

Myogenic c-kit-BMCs were characterized by enrichment of the c-kit transcript (Fig. 5a; Supplementary Dataset S1). Although we cannot exclude that changes in c-kit level occurs in vitro, a high variability of expression of the receptor tyrosine kinase is commonly observed in freshly isolated BMCs positive for c-kit. Significant differences in brightness of the fluorochrome conjugated with the c-kit antibody are visible by flow-cytometry (Supplementary Fig. S3). The upregulation of c-kit was accompanied by differences in the expression of the members of the transmembrane 4 superfamily (TM4SF), CD63 and CD81. The TMSF4 complex is physically associated with the c-kit receptor and controls its tyrosine kinase activity and the sensitivity of the response to stem cell factor (SCF).^18^ CD81 was downregulated while CD63 was markedly upregulated in myogenic c-kit-BMCs. Importantly, CD63 recognizes a class of c-kit-positive CSCs with a high tendency to differentiate into cardiomyocytes.^19^ This observation, together with the documentation that cardiomyocytes, ECs and fibroblasts express CD63, (http://www.proteinatlas.org/ENSG00000135404-CD63/tissue) provides a molecular link between c-kit-BMCs and specialized cardiac cells. The expression pattern of CD63 and CD81 in myogenic c-kit-BMCs appeared to promote c-kit signaling as documented by the upregulation of the target gene MITF (Fig. 5a; Supplementary Dataset S1). MITF supports the adhesion of HSCs to stromal cells, homing and long-term repopulating property^20^ and is expressed in c-kit-positive CSCs and their myocyte derivatives.^21^ These observations suggest that MITF may favor the engraftment of c-kit-BMCs in the damaged myocardium and their commitment to the myocyte fate, through the activation of the promoters of myosin light-chain 1a and GATA4.^22^

Survival and engraftment of stem cells are critical for the initiation of the reparative process. Subsequently, proliferation and translocation of the homed viable cells to the infarcted myocardium require the activation of an anti-adhesive and pro-migration molecular program. In analogy with c-kit-positive CSCs,^23^ the enrichment for Pim1 kinase in myogenic c-kit-BMCs may oppose cell death and stimulate cell replication (Fig. 5b). Upregulation of cathepsin A, C, and D, which encode proteins that degrade the extracellular matrix, may favor the colonization and movement of the injected c-kit-BMCs.^24^ A similar pro-invasive effect may be achieved by the enrichment of Sema 4D, a surface and soluble protein that binds to the plexin 1 receptor, triggering the kinase activity of c-Met.^25^ Moreover, downregulation of the microtubule destabilizer stathmin (Fig. 5b; Supplementary Dataset S1) may enhance the duplication and motility of c-kit-BMCs.^26^

DEGs were subjected to Gene Ontology (GO) for their functional classification; the top 10 terms of GO functional analysis are shown for each category in Fig. 5c (see also Supplementary Dataset S1). Based on the KEGG pathway database, an additional enrichment test was conducted. The enrichment heatmap involved six major biological processes comprising several subcategories (Fig. 5d; Supplementary Figure S4), in which similarly and differentially expressed gene networks were identified by applying the modified fisher’s exact text followed by Bonferroni and FDR analysis (Supplementary Dataset S1).

The significant pathway modules related to surface proteins included the c-kit receptor (CD117) and the epitopes CD13, CD41, CD42, CD55, CD59, and CD124, which were upregulated in the myogenic c-kit-BMCs (Supplementary Fig. S5). Conversely, CD11b, CD44 and CD114 were downregulated in this cell class. The combination of these epitopes indicates that myogenic c-kit-BMCs do not possess a specific hematopoietic and mesenchymal cell subtype lineage.

The pathways involved in stem cell fate (Wnt and HIF-1 signaling) and cell growth and death (PI3K-Akt signaling) converged on the upregulation of PKC-α and PKC-β in myogenic c-kit-BMCs (Supplementary Fig. S6–S8); these signaling cascades favor the commitment of embryonic and adult progenitors into the cardiomyocyte lineage.^27^ TGFβ receptors constituted significantly upregulated pathway modules within the HIPPO, MAPK, and FOXO signaling; TGF-β induces reprogramming of c-kit-BMCs into immature cardiomyocytes that express sarcomeric and gap junctional proteins.^28^

The upregulation of Hes 1-5 in myogenic c-kit-BMCs (Supplementary Fig. S9) reflected the activation of the Notch receptor, which induces the commitment of CSCs to the myocyte lineage and defines the size of the compartment of cycling myocytes in vitro and in vivo.^29^ This function of Notch1 involves the transactivation of Nkx2.5, which drives myocyte specification of endogenous c-kit-positive CSCs.^29^ p21^Cip1^ is a significant pathway module in multiple networks (ERBB, HIF-1, Foxo, cell cycle, PI3K-AKT) and may be involved in the transient activation of growth arrest in myogenic c-kit-BMCs and the repair of DNA damage^30^ induced by stress stimuli present in the infarct border zone.

These findings suggest that myogenic clonal c-kit-BMCs are characterized by the enrichment of genes, which confer to this cell subset a biological advantage for the regeneration of the injured myocardium. The gene cluster present at the level of the plasma membrane offers the opportunity to isolate subpopulations of c-kit-BMCs with cardiopoietic properties and may form the basis for future studies addressing the reparative response of c-kit-BMCs following severe forms of myocardial damage and heart failure.

### Multicolor clonal tracking of c-kit-BMCs and their progeny

**Fig. 6:**
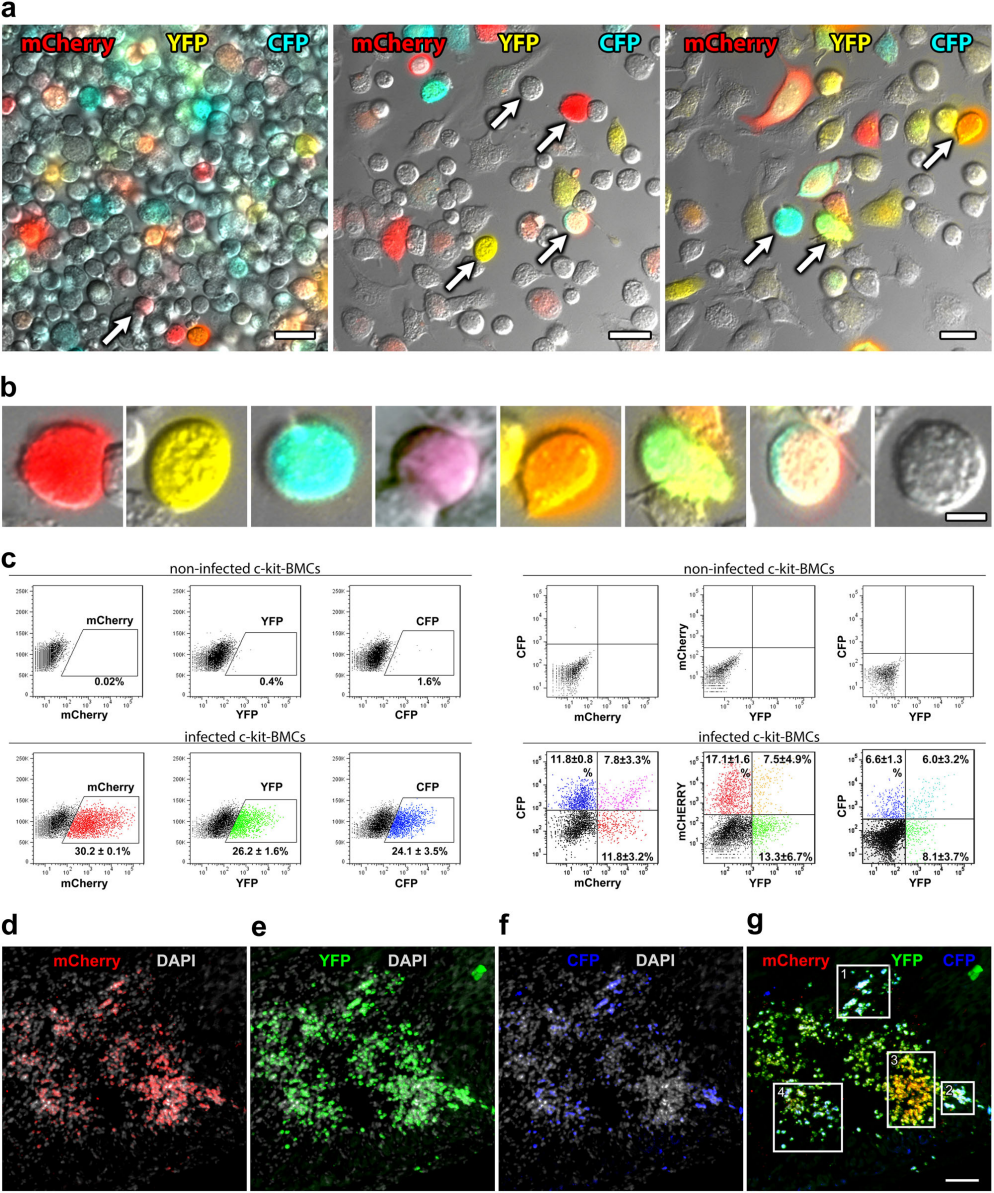
c-kit-BMCs express three fluorescent reporter genes in vitro and in vivo. **a** Low power magnification images illustrating native fluorescence of c-kit-BMCs transduced with three lentiviruses carrying eCFP (blue), mCherry (red) or eYFP (yellow). Scale bars = 20 µm. Arrows indicate the cells illustrated at higher magnification in panel b. **b** Individual c-kit-BMCs show the primary colors, i.e., red, yellow and cyan, and their multiple combinations. Scale bars = 5 µm. **c** Scatter plots documenting the detection of YFP, CFP or mCherry and their combinations in c-kit-positive cells by flow-cytometry. Non-infected c-kit-BMCs were used as negative control (upper panels). **d–g** 4 days after coronary artery occlusion and the delivery of red-green-blue (RGB) marked c-kit-BMCs, an area of the infarcted myocardium is replaced by cells positive for mCherry (**d**, red), YFP (**e**, green), and CFP (**f**, blue). The 4 rectangles in the merged panel (**d**) delineate clusters of cells uniformly labeled: clusters 1 and 2 are composed of cells predominantly white (red, green and blue together = white); cluster 3 is composed of cells predominantly yellow (red and green together = yellow); and cluster 4 is composed of cells predominantly turquoise (green and blue together = turquoise). Sections **d**–**g** were examined by epifluorescence microscopy. Scale bar = 200 µm

Viral tagging and RNA sequencing are coupled with dissociation of cardiac tissue and cells, precluding the possibility to observe myocardial regeneration in situ. We felt unnecessary to reiterate in the current study aspects which were addressed repeatedly in our previous work, including the morphological characterization and quantitative assessment of the cardiac repair process. Immunolabeling techniques and microscopic analyses were introduced to perform a multicolor lineage tracing^14^ of c-kit-BMC fate in the infarcted heart. The combination of fluorescent protein signals of distinct colors is relevant for the recognition of clonally related cells. Three lentiviral vectors carrying, respectively, mCherry (red), YFP (yellow), and CFP (cyan) fluorescent protein^14^ were employed to infect c-kit-BMCs. Each color and their mixture were evaluated in cultures of c-kit-BMCs by examining native red, yellow and cyan fluorescence with an epifluorescence microscope (Fig. 6a, b). These qualitative observations were complemented with flow cytometry to evaluate quantitatively labeled c-kit-BMCs (Fig. 6c). Based on the additive color theory, we assigned the 3 primary colors, i.e., red, green, and blue, to mCherry, YFP, and CFP, respectively. These basic colors give rise to secondary colors formed by the blend of red, green, and blue.^14^ Eight separate cell categories were detected: they included c-kit-BMCs transduced with only one of each of the 3 viral vectors; these cells showed red fluorescence in 24.3% of the cases, green in 18%, and blue in 15.2%. Three more classes of cells showed the combination of red and green, i.e., yellow: 2.8%; red and blue, i.e., violet: 3.0%; and green and blue, i.e., turquoise: 3.1%. One cell category was labeled by red, green, and blue, i.e., white: 1.9%; and one was not labeled, 31.8%.

Following acute myocardial infarction, c-kit-BMCs infected with the 3 lentiviruses were delivered to the border zone, and the animals were sacrificed 4–7 (*n* = 12) and 14–21 (*n* = 13) days later. At 4–7 days, areas of myocardial regeneration, varying in size, were identified within the infarcted region of the left ventricular (LV) wall (Supplementary Fig. S10). The foci of tissue repair, examined by epifluorescence microscopy, were characterized by multiple clusters of uniformly colored cells, indicating their origin from a single c-kit-BMCs (Fig. 6d–g).

**Fig. 7:**
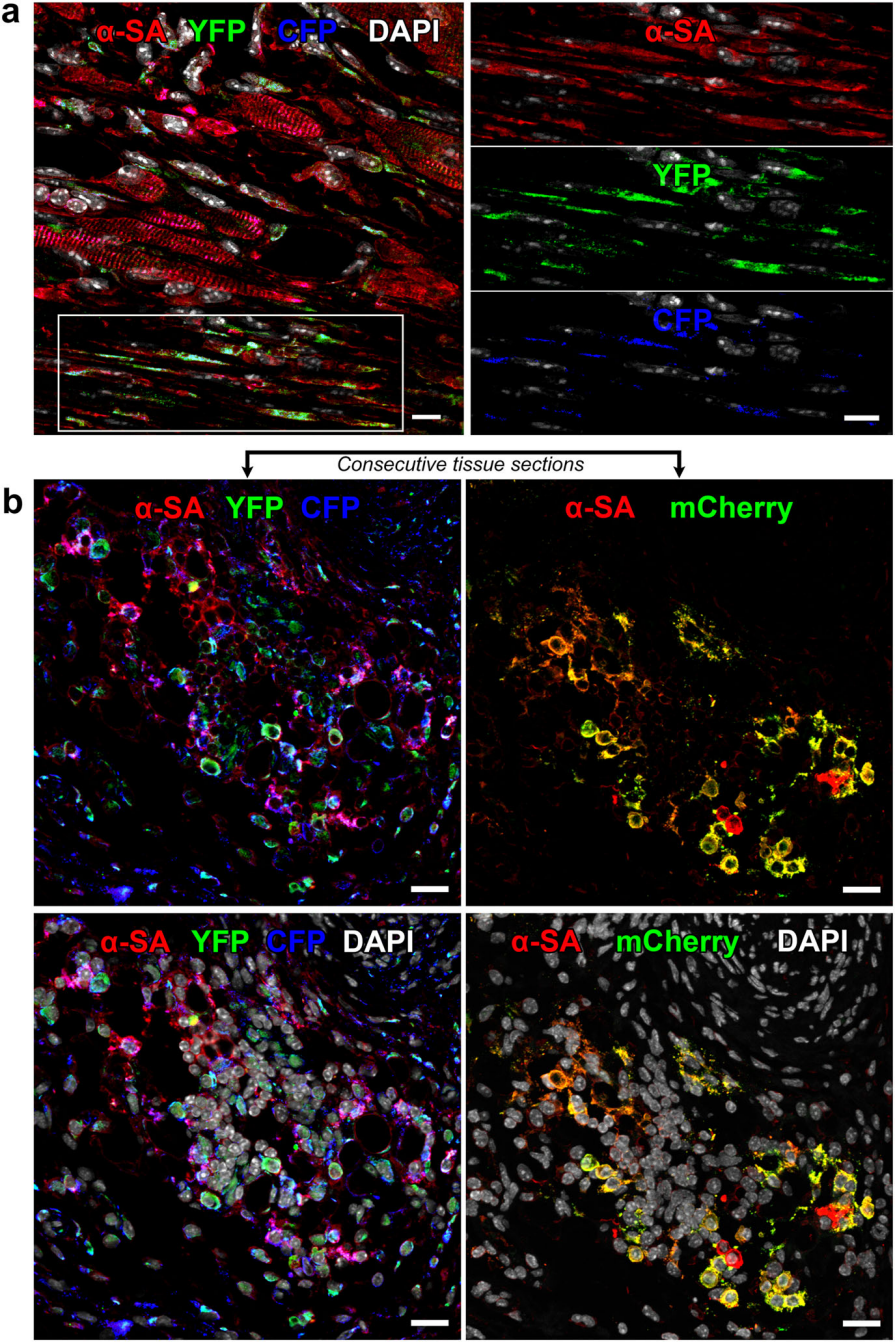
c-kit-BMCs acquire the cardiomyocyte lineage. **a** The white rectangle in the lower part of the left panel comprises regenerated cells located in proximity of the spared myocardium; these cells are tagged by YFP (green) and CFP (blue); green and blue together = turquoise and express the contractile protein α-SA (red). Labeling for α-SA, YFP and CFP is shown separately in the three right panels. Scale bars = 10 µm. **b** Group of developing cardiomyocytes shown in two consecutive tissue sections (upper panels) to detect the three tags. The upper left panel shows the co-localization of α-SA (red), YFP (green) and CFP (blue), and the upper right panel shows the co-localization of α-SA (red) and mCherry (assigned color: green). The two lower panels illustrate the same images with nuclei stained by DAPI (white). Scale bars = 20 µm

To determine the fate of the formed cells, tissue sections were stained with markers specific for cardiomyocytes and vascular cells. At 4–7 days after coronary occlusion and cell delivery, the infarcted region was largely replaced by patches of homogeneously colored cardiomyocytes derived from c-kit-BMCs carrying mCherry, YFP, CFP, or their combination (Fig. 7). Consecutive tissue sections were evaluated to discriminate groups of cardiomyocytes carrying a single or multiple vectors. Consistent with the findings obtained by viral gene tagging, c-kit-BMCs regenerated coronary vessels of different size distributed throughout the reconstituted myocardium (Supplementary Fig. S11).

**Fig. 8:**
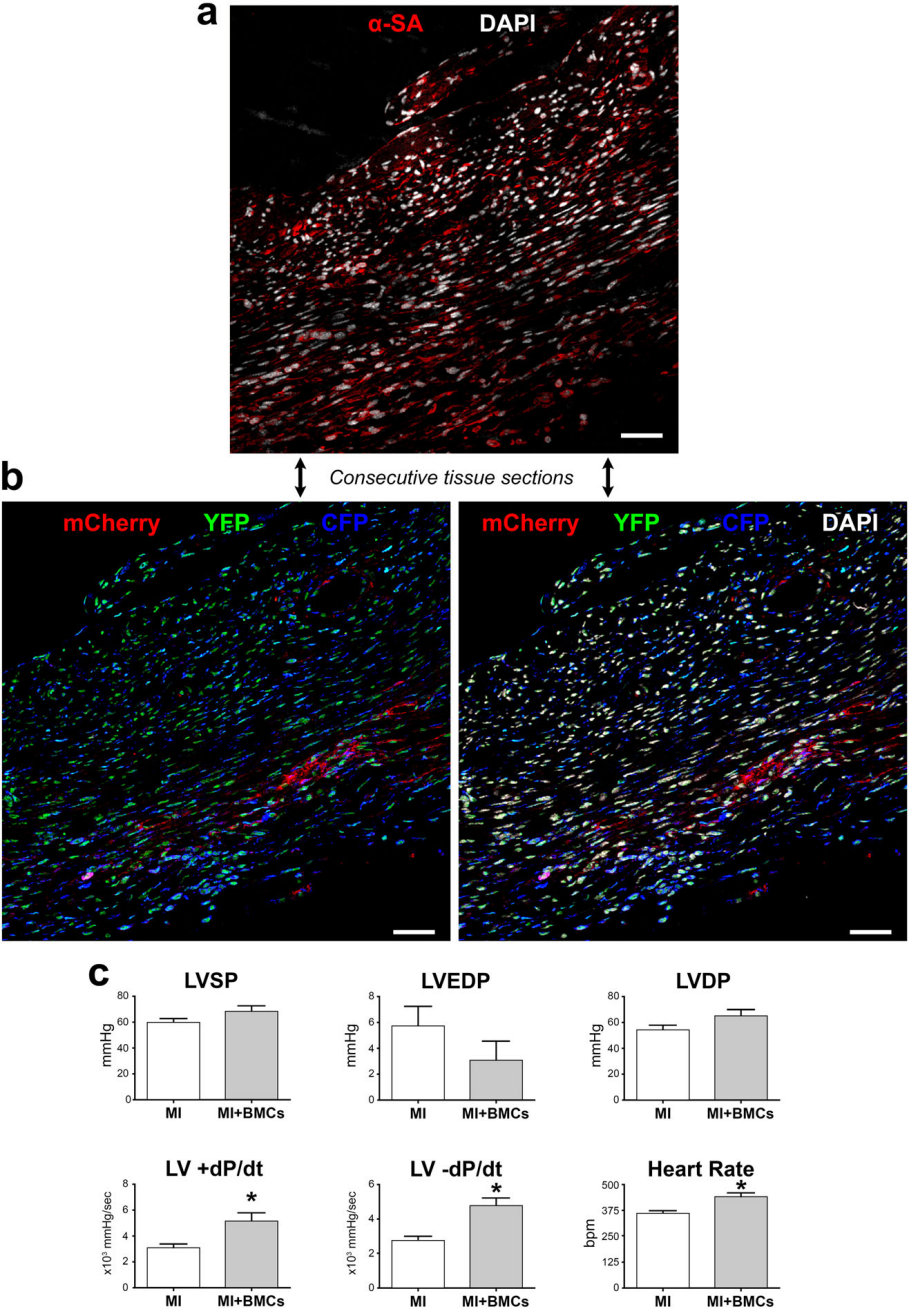
Differentiation of c-kit-BMCs into cardiomyocytes. **a**, **b** Consecutive tissue sections at 15–21 days after infarction. The regenerated myocytes are positive for α-SA (**a**, red), for mCherry (**b**, red), YFP (**b**, green) and CFP (**b**, blue). Labeling of DAPI (white) is shown in the upper and lower right panels. Scale bars = 100 µm. **c** Measurements of ventricular pressures and dP/dt in untreated infarcts (MI: *n* = 11) and cell-treated infarcts (MI + BMCs: *n* = 8). *P < 0.05

At 14–21 days after infarction and cell delivery, considerable areas of the infarcted LV were replaced by small fluorescently labeled cells, expressing α-sarcomeric actin (α-SA) and GATA4 (Fig. 8a, b; Supplementary Fig. S12). Foci of cardiac repair were composed of large clusters of cells, which showed a rather consistent distribution of one, two, or three colors. The measurement of left ventricular (LV) hemodynamics at two weeks showed that the delivery of c-kit-BMCs to the infarcted heart resulted in a better preservation of LV systolic pressure (LVSP), a smaller elevation in LV end-diastolic pressure (LVEDP), a higher value of LV developed pressure (LVDP) and a significant increase in positive and negative dP/dt (Fig. 8c). This analysis included 11 untreated and 8 cell-treated infarcts. Thus, c-kit-BMCs repair the infarcted heart by forming, in a coordinated manner, cardiomyocytes and coronary vessels, which results in an amelioration of cardiac performance.^31^

## Discussion

The results of the present study indicate that the compartment of c-kit-BMCs is diverse and only a subset of these cells possesses the inherent ability to home to the injured heart and commit to the main cardiac lineages. In contrast, some c-kit-BMCs remain undifferentiated and retain their original identity. The documentation of the functional multiplicity of c-kit-BMCs has required the implementation of molecular-genetic strategies, which have provided a different appreciation of the plasticity of c-kit-BMCs. The heterogeneity of stem cells can only be resolved by introducing single-cell-based assays; viral gene tagging and clonal marking provided a genetic confirmation that individual c-kit-BMCs engraft within the infarct and become a relevant component of the cardiac repair process. The recognition that cardiomyocytes, vascular ECs, fibroblasts, and c-kit-BMCs isolated from infarcted treated hearts have common sites of viral integration provides powerful evidence in support of BMC transdifferentiation.

Despite the shared expression of the c-kit receptor tyrosine kinase, apparently similar c-kit-BMCs behave differently following transplantation in vivo. Cardiomyogenesis was utilized here as readout for the retrospective documentation of the ability of c-kit-BMCs to cross lineage boundaries. The evaluation of clones derived from single c-kit-BMCs allowed the identification of rare stem cell subsets, which are lost in population-based studies where they may be viewed as outliers or may be absorbed by larger clusters of cells.^32^

Understanding the bases of the divergent results obtained in several laboratories following the injection of c-kit-BMCs within the infarcted heart is challenging. The inconsistencies in the reported findings may be related to a variety of factors including technical difficulties, variability in the phenotype of the employed c-kit-BMCs and methodologies of analysis. The evidence of transdifferentiation of c-kit-BMCs was tested by Drs. Wagers and Weissman, which failed to reproduce our early results.^6^ 10 days after myocardial infarction and cell delivery, clusters of GFP-positive cells negative for myocyte and vascular antigens but positive for the myeloid marker Gr-1 were detected, suggesting that BMCs adopt the mature hematopoietic fate in the injured heart. However, this conclusion was reached on the basis of 2 infarcted mice,^6^ which were supposedly properly infarcted and injected with lineage-negative c-kit-BMCs comparable to those used in our laboratory.^1^ In fact, additional mice were injected with different BMC pools or were joined in parabiosis, an artificial in vivo system that cannot be compared with the direct transplantation of cells at the site of injury.^6,33^

Studies performed in c-kit mutant mice have suggested that c-kit-BMCs promote angiogenesis in the ischemic myocardium, but do not contribute to myocyte formation.^34^ However, the expression of the c-kit receptor with intact tyrosine kinase activity has been shown to be critical for the differentiation of BMCs to cardiomyocytes in vitro.^35^ More recently, a double transgenic mouse for genetic lineage mapping was introduced to determine whether c-kit-BMCs generate cardiomyocytes following injury. In this model, all cardiomyocytes express β-galactosidase (β-gal) but, after a pulse of tamoxifen, myocytes switch to GFP expression, as a result of Cre-mediated DNA recombination driven by the α-myosin heavy chain promoter (α-MHC). GFP expression was restricted to a category of α-MHC-positive cells, resulting in cardiac chimerism with co-existence of β-gal-labeled (inactive-Cre) and GFP-labeled (active-Cre) cells. The changes in the proportion of β-gal- and GFP-positive cardiomyocytes were used to define whether the contractile cells originated from c-kit-BMCs (myocytes negative for β-gal and GFP), endogenous progenitors (myocytes positive for β-gal only) or pre-existing cardiomyocytes (myocytes positive for GFP only).^36^ However, this mouse model does not answer this critical question.

The reason why a group of cardiomyocytes does not express the reporter gene is unclear. The presence or absence of GFP is likely dictated by inherent features of the two subsets of differently-labeled myocytes and the phenotypical and functional heterogeneity of adult myocytes. Unfortunately, fate mapping is a population-based strategy, which offers reliable evidence only when pools of nearly identical cells are considered, a process that does not occur in nature. This limitation can be easily overcome by isolating labeled and unlabeled myocytes and analyzing their characteristics. The assessment of size, shape, nuclear number, electromechanical properties and calcium transient of GFP-positive and GFP-negative cardiomyocytes can clarify whether immature, fully-developed and senescent cells are asymmetrically-segregated in the two differently labeled populations.

Surface markers that permit the prospective isolation of functionally homogenous stem cell classes have not been discovered yet. Information in this regard has been obtained by RNA-sequencing of myogenic c-kit-BMCs, which possess a molecular signature that comprises a network of transcripts favoring engraftment, survival and migration in the hostile environment of the injured myocardium as well as the acquisition of the cardiogenic fate. The panel of membrane epitopes found to be upregulated and downregulated in myogenic c-kit-BMCs offers the opportunity to isolate highly plastic cells. Similarly, the recognition of uniformly colored clusters of specialized cells demonstrate the clonal expansion and commitment of single c-kit-BMCs in vivo. These findings are consistent with the results obtained by viral genome integration which, together, reveal the multi-clonal origin of myocardial reconstitution. Importantly, this process attenuated the alterations in ventricular performance of the infarcted heart. Although modest, the therapeutic effect of c-kit-BMCs could be appreciated in spite of the immature phenotype of the newly-formed cardiomyocytes. The measurement of the number of myogenic and vasculogenic clones in situ requires highly sophisticated imaging and labeling technology capable of defining clonal brightness and chromatic stability and physically pooling cells based on clonal chromatic mode and spread.^37^ This quantitative analysis was beyond the scope of our study and is not critical for the questions addressed in this work.

The possibility that c-kit-BMCs may fuse with recipient cardiomyocytes prior to myocardial regeneration cannot be excluded by viral gene tagging. But, the fetal characteristics of newly-formed cardiomyocytes and the previous analysis of this process^31^ make this an unlikely event. In mice joined by parabiosis and sharing a common circulatory system, GFP-labeled blood cells migrate from the BMC-transplanted parabiont to the parabiont subjected to myocardial infarction.^38^ Although at very low rate, these cells fuse with cardiomyocytes and contribute to cardiac repair. The identity of fusing BMCs has been defined by adoptive transfer of these cells to the infarcted heart. Studies in which male cells were injected in female infarcted mice have excluded that lineage negative c-kit-BMCs act as partners of cardiomyocytes in the formation of heterokaryons.^31,36^ However, Gr1-positive myeloid progenitors have a great proficiency to fuse with the recipient cardiomyocytes;^39,40^ this is consistent with the intrinsic ability of inflammatory cells to coalesce and form giant multinucleated cells. In a single report, the injection of BMCs purified for c-kit was found to be coupled with the formation of BMC-cardiomyocyte hybrids. However, the engrafted BMCs expressed uniformly CD45 together with myeloid and lymphoid antigens.^41^ Thus, fusion seems to be the preferential mechanism of action of migrating committed BMCs and inflammatory cells after infarction.^42^

Thus far, only BM-MNCs, CD34-positive cells and mesenchymal stromal cells have been employed clinically.^2^ Our findings indicate that c-kit-BMCs may be considered as an alternative form of cell therapy for the failing heart in view of the limited beneficial effects observed with BM-MNCs experimentally^43^ and clinically.^2^ None of the clinical trials performed in the last several years has employed c-kit-BMCs. Moreover, the therapeutic efficacy of c-kit-BMCs and resident c-kit-positive CSCs for myocardial repair has never been compared. Based on a microarray assay, these two classes of c-kit-positive cells have a highly distinct transcriptional profile,^44^ but when delivered to the same microenvironment appear to acquire similar functional characteristics. Bioinformatic analysis of published RNA-sequencing data may provide important information on the signaling pathways, which are more relevant to cardiomyogenesis. The molecular differences may be attenuated within the damaged myocardium and bone marrow-derived and cardiac-derived progenitor cells may act similarly in reconstituting partly the integrity of the tissue. In analogy to c-kit-BMCs, c-kit-CSCs have been found recently to operate via paracrine mechanisms^45^ and/or to act as source of cardiomyocytes and coronary vessels.^46^ It is not surprising that despite the accurate execution of sophisticated methodologies employed by different research groups diverse results have been obtained. The approach implemented in the current study may help clarifying these apparent discordant observations.

## Materials and methods

### Detection of sites of viral integration in cardiac cells

#### Culture and lentiviral infection of c-kit-BMCs

The bone marrow was harvested from the femurs and tibias of C57Bl/6 mice at 2 months of age.^1,31^ Lysis of erythrocytes was obtained by incubating BMCs with BD Pharm Lyse™ (Beckton Dickinson) for 15–20 min at room temperature. BM-MNCs were washed with PBS containing 0.5% bovine serum albumin (BSA) and 2 mM EDTA (Gibco). Cells were re-suspended in washing buffer and incubated with mouse monoclonal CD117-microbeads (130-091-224; Miltenyi) for 15 min at 4 °C. c-kit-BMCs were enriched by magnetic-activated cell sorting (MACS) and plated in non-coated dishes for 2 days. Cells were cultured with Iscove’s Modified Dulbecco’s Medium (IMDM, Invitrogen), supplemented with thrombopoietin (20 ng/ml), interleukin-3 (20 ng/ml), interleukin-6 (40 ng/ml), Fms-related tyrosine kinase 3 ligand (10 ng/ml), stem cell factor (50 ng/ml), and 10% fetal bovine serum (FBS) in the presence of penicillin and streptomycin.^47^ GFP-lentiviral supernatant was added to retronectin-coated (Takara) dishes. Floating c-kit-BMCs were then transferred, 2 × 10^5^ cells/dish, and expanded for 3 days.

#### Myocardial infarction and transplantation of GFP-labeled c-kit-BMCs

All protocols were approved by the Institutional Animal Care and Use Committee (IACUC) of Brigham and Women’s Hospital. Animals received humane care in compliance with the Guide for the Care and Use of Laboratory Animals as described by the Institute of Laboratory Animal Research Resources, Commission on Life Sciences, National Research Council. Myocardial infarction was induced in anesthetized (isoflurane 1.5%) female C57Bl/6 mice at 3 months of age as previously described.^1,31,48^ Shortly after coronary artery ligation, FACS-sorted GFP-labeled c-kit-BMCs, 1 × 10^5^ per heart, were injected in four different sites of the region bordering the infarct. Animals were sacrificed two weeks later.

#### Enzymatic dissociation and isolation of cardiac cells

At sacrifice, hearts were enzymatically digested with protease and collagenase type II (Worthington) to obtain a single cell suspension.^48^ Hearts were excised and placed on a stainless steel cannula for retrograde perfusion through the aorta. The solutions were supplements of modified commercial MEM Joklik (Sigma). HEPES/MEM contained 117 mM NaCl, 5.7 mM KCl, 4.4 mM NaHCO_3_, 1.5 mM KH_2_PO_4_, 17 mM MgCl_2_, 21.1 mM HEPES, 11.7 mM glucose, amino acids, and vitamins, 2 mM L-glutamine, 10 mM taurine, and 21 mU/ml insulin and adjusted to pH 7.2 with NaOH. Re-suspension medium was HEPES/MEM supplemented with 0.5% BSA, 0.3 mM calcium chloride, and 10 mM taurine. The cell isolation procedure consisted of four main steps. (1) Calcium-free perfusion: blood washout and collagenase type II-perfusion of the heart was carried out at 34 °C with HEPES/MEM gassed with 85% O_2_ and 15% N_2_. (2) Mechanical tissue dissociation: after the heart was removed from the cannula, the collagenase-perfused myocardium was minced and subsequently shaken in resuspension medium containing collagenase. (3) Myocyte separation: cells were centrifuged at 30×*g* for 3 min. This procedure was repeated four to five times. Myocytes were recovered from the pellet and the supernatant was collected. (4) Separation of small cardiac cells: cells were obtained from the supernatant and sorted by FACS with antibodies recognizing rat monoclonal c-kit (553356 (APC), 553354 (FITC) or 561075 (PE); BD Pharmingen), rat monoclonal CD31 (561410 (PE-Cy7); BD Pharmingen), and rat monoclonal Thy1.2 (553007 (PE); BD Pharmingen). ECs were positive for CD31 and negative for Thy1.2 and c-kit; fibroblasts were positive for Thy1.2 and negative for CD31 and c-kit; and BMCs were positive for c-kit only.^48^

#### Purity of the isolated populations of cardiac cells

The purity of the cell preparations was documented by RT-PCR and by immunolabeling. For qRT-PCR, total RNA was isolated from myocytes and FACS-sorted c-kit-BMCs, ECs and fibroblasts with RNeasy mini kit (Qiagen). Total RNA was converted to cDNA using High Capacity cDNA synthesis kit (Applied Biosystems). qRT-PCR was performed on 7300 Real Time PCR System (Applied Biosystems) using 1/20th of the cDNA per reaction. Primers were designed from available mouse sequences using the primer analysis software Vector NTI (Invitrogen). Transcripts of α-cardiac myosin heavy chain (*Myh6*), CD31, collagen type III, α-1 (*Col3a1*), c-kit and the housekeeping gene β-2 microglobulin (*B2M*) were measured. Mouse myocardium was used as control. The PCR-reaction included 1 μl template cDNA, 500 nM forward and reverse-primers in a total volume of 20 μl. Cycling conditions were as follows: 95 °C for 10 min followed by 35 cycles of amplification (95 °C denaturation for 15 s, and 60 °C combined annealing/extension for 1 min). Primers were as follows:

c-kit-Forward: 5′- GGA GAT CCG CAA GAA TAG ACT CGT AC -3′

c-kit-Reverse: 5′- CTT TGT GAT CCG CCC GTG AGT -3′

Myh6-Forward: 5′- ACC AAC CTG TCC AAG TTC CG -3′

Myh6-Reverse: 5′- TAT TGG CCA CAG CGA GGG TC -3′

CD31-Forward: 5′- AGC TGC TCC ACT TCT GAA CTC -3′

CD31-Reverse: 5′- TCA AGG GAG GAC ACT TCC AC -3′

Col3a1-Forward: 5′- GGT GAC AGA GGA GAA ACT GG -3′

Col3a1-Reverse: 5′- ATG TGG TCC AAC TGG TCC TC -3′

B2M-Forward: 5′- CTC GGT GAC CCT GGT CTT TC -3′

B2M-Reverse: 5′- TTC AGT ATG TTC GGC TTC CC -3′

RT-PCR products were run on 2% agarose/1x TAE gel and bands of distinct molecular weight were identified.

For immunolabeling, isolated cardiomyocytes and FACS-sorted ECs and fibroblasts were fixed in suspension with 4% paraformaldehyde. Aliquots of cells were deposited on slides and labeled with antibodies recognizing mouse monoclonal α-SA (A2172; Clone 5C5; Sigma) or sheep polyclonal von Willebrand factor (vWF; ab11713; Abcam), and goat polyclonal procollagen (Pro-Col; Clone Y18; sc-8787; Santa Cruz Biotechnology). Nuclei were stained by DAPI. The fraction of cells positive for lineage markers was then determined by fluorescent microscopy.

#### Identification of proviral integrants in the mouse genome

Each integration site corresponds to a distinctive genomic sequence, which was detected on the assumption that a restriction enzyme (RE) cleavage site was present at a reasonable distance (20–800 bp) from long terminal repeats (LTRs) flanking the viral genome. Following the cleavage of the genomic DNA with the RE, DNA products were self-ligated to produce circularized DNA.^9,48,49^ Different primers and distinct RE were employed to optimize the methodology of detection of the viral integration site. This step created a genomic sequence of variable length due to the random location of the RE site within the lentiviral flanking region. Since the unknown lentiviral flanking region was entrapped between two known sequences, it was possible to amplify the viral integration site by PCR.

Genomic DNA was extracted separately from populations of cardiomyocytes, ECs, fibroblasts and c-kit-BMCs with QIAamp DNA Mini Kit (QIAGEN) isolated from 8 cell-treated hearts. The extracted DNA was digested with Taq I (New England Biolabs) for 2 h at 65 °C. The enzyme was heat-inactivated at 80 °C for 25 min. Aliquots of samples were run on agarose gel to confirm digestion. To circularize DNA fragments, samples were incubated with 10 µl Quick T4 DNA Ligase (New England Biolabs) in a total reaction volume of 200 μl and kept at room temperature overnight. Phenol/chloroform and chloroform extractions were then performed. After 2-propanol precipitation, DNA was re-linearized with Hind III (10 U). The protocol utilized for the recognition of the integrated provirus corresponds to an inverse PCR, which is the most sensitive strategy for the amplification of unknown DNA sequences that flank a region of known sequence.^49^ The primers are oriented in the reverse direction of the usual orientation and the template is a restriction fragment that has been ligated to be self-circularized. One round of PCR and two additional nested PCR were performed utilizing AccuPrime Pfx SuperMix (Invitrogen). At each PCR step, samples were diluted 1:2,500. The PCR primers employed in the first (1st) and second (2nd) amplification rounds were designed in the region of LTR which is commonly located at the 5′- and 3′- side of the lentiviral genome. The PCR primers employed in the third round (3rd) were specific for the 3′-side of the site of integration. In all cases, primers were oriented in the opposite direction.

### First round PCR

eGFP-X: GGTTCCCTAGTTAGCCAGAGAGC (23nt)

eGFP-Y: GAGTGCTTCAAGTAGTGTGTGC (22nt)

95 °C for 5 min; 40 cycles of 95 °C for 15 s, 55 °C for 30 s, 68 °C for 70 s; 68 °C for 2 min.

### Second round PCR

eGFP-M: AGCAGATCTTGTCTTCGTTGGGAGTG (26nt)

eGFP-Z: CCGTCTGTTGTGTGACTCTGGTAA (24nt)

Same cycling condition as above but with 25 cycles.

### Third round PCR

eGFP-F: 5′- CATTGGTCTTAAAGGTACCGAGCTCG -3′

eGFP- L: 5′- GATCCCTCAGACCCTTTTAGTCAGTG -3′

Same cycling condition as the second round.

Taq polymerase-amplified PCR products were inserted into the plasmid vector pCR4-TOPO using the TOPO TA Cloning Kit (Invitrogen). Subsequently, chemically competent TOP10 *E. coli* cells were transformed with the vector carrying the PCR products. The transformation mixture was spread on agar plates and incubated overnight at 37 °C. Ten to twenty colonies from each plate were expanded in 10 ml LB medium containing ampicillin. The amplified constructs were extracted with the QIAGEN Plasmid Purification Mini-Kit, digested with EcoR I, and run on agarose gel. Bands of different molecular weight were identified in 7 of the 8 hearts examined. DNA sequencing was performed to verify the presence of viral integration sites.

### Red, green, and blue (RGB) marking of c-kit-BMCs

#### Culture and lentiviral infection of c-kit-BMCs

c-kit-BMCs were cultured (see above) and concurrently infected with three lentiviral vectors carrying distinct fluorochromes.^14,50,51^ The following viruses were employed: (1) EX-mChER-Lv105 - vector with mCherry for pReceiver-Lv105, which corresponds to an HIV-based lenti-vector with a CMV promoter and puromycin selection marker; (2) EX-eYFP-Lv102 - vector with enhanced yellow fluorescent protein (eYFP) for pReceiver-Lv102, which corresponds to an HIV-based lenti-vector with a CMV promoter, N-FLAG tag and puromycin selection marker; and (3) EX-eCFP-Lv107 - vector with enhanced cyan fluorescent protein (eCFP) for pReceiver-Lv107, which corresponds to an HIV-based lenti-vector with a CMV promoter, N-Myc tag.

#### In vitro detection of fluorescent markers

Native fluorescence of mCherry, eYFP, and eCFP in c-kit-BMCs was established by epifluorescence microscopy. The presence of the three primary colors and their combinations was detected in the majority of c-kit-BMCs. The quantitative analysis of the proportion of c-kit-BMCs infected by one, two or three vectors was performed by FACS, utilizing native fluorescence.

#### Myocardial infarction and transplantation of RGB-labeled c-kit-BMCs

Myocardial infarction was induced as described above. Acutely after coronary artery ligation, 1 × 10^5^ c-kit-BMCs infected with the three lentiviruses carrying mCherry, YFP, or CFP were injected at 4 sites in the region bordering the infarct.^31,48^ Animals were sacrificed 4–7 and 14–21 days later. Briefly, the abdominal aorta was cannulated with a polyethylene catheter filled with heparin–sodium injection solution (1000 units/ml). In rapid succession, the heart was arrested in diastole by injection of cadmium chloride (100 mM), and perfusion with phosphate buffer was conducted for ~3 min. The thorax was then opened, and the right atrium was cut to allow drainage of blood and perfusate. The heart was fixed by perfusion with 10% phosphate-buffered formalin. After fixation, the heart was dissected, and sections from the base and mid-portion of the left ventricle were examined.^1,31,48^ Immunolabeling was performed with: mouse monoclonal mCherry antibody (ab125096; Clone 1C51; Abcam) for the detection of mCherry; rabbit polyclonal DDDDK tag antibody (ab21536; Abcam) for the detection of the N-FLAG tag in the eYFP lentivirus; and chicken polyclonal Myc tag antibody (ab172; Abcam) for the detection of the N-Myc tag in the eCFP lentivirus. Cardiomyocytes were identified by antibodies recognizing goat polyclonal Nkx2.5 (sc-8697; Santa Cruz Biotechnologies), goat polyclonal GATA4 (sc-1237; Santa Cruz Biotechnologies), mouse monoclonal α-sarcomeric actin (A2172; Clone 5C5; Sigma), or rabbit polyclonal Troponin (ab47003; Abcam). Vascular ECs and SMCs were detected, respectively with mouse monoclonal Anti-Actin, a-SMA (A5228; Clone 1A4; Sigma), sheep polyclonal vWF (ab11713; Abcam) and goat polyclonal CD31/PECAM-1 (AF3628; R&D). Rabbit polyclonal anti-connexin 43 (C6219; Sigma) was employed to illustrate this gap junctional protein.

At 14 days after infarction, LV hemodynamics loops were obtained in untreated (*n* = 11) and cell treated (*n* = 8) mice. The parameters were obtained in the closed-chest preparation with a MPVS-400 system for small animals (Millar Instruments) equipped with a PVR-1045 catheter.^52,53^ Mice were intubated and ventilated (MiniVent Type 845; Hugo Sachs Elektronik-Harvard Apparatus, GmbH, March, Germany) with isoflurane anesthesia (isoflurane, 1.5%); the right carotid artery was exposed and the pressure transducer was inserted and advanced in the LV cavity. Data were acquired with LabChart (ADInstruments) software.

### Clonal assay for the identification of myogenic c-kit-BMCs

#### Preparation of c-kit-BMC clones and in vivo transplantation

Freshly isolated c-kit-BMCs were infected with a lentivirus carrying GFP. Subsequently, c-kit-positive GFP-positive BMCs were FACS-sorted and seeded at limiting dilution in Methocult-coated wells (3 × 10^3^ per well). Over a period of 10 days, small colonies derived from individual BMCs were observed. Cells were further expanded and the expression of c-kit and GFP was determined; 15 clones were employed for in vivo assays and DNA and RNA extraction. A total of 1 × 10^5^ cells, i.e., 2 × 10^4^ from each of 5 clones, were injected in the border zone of acutely infarcted mice, and the animals were sacrificed 21 days later for the detection of the site of viral integration in regenerated cardiomyocytes. Cardiomyocytes were collected by enzymatic digestion as described above. Additionally, the site of integration in c-kit-positive GFP-positive BMCs formed in each clone was determined to establish the lineage relationship between specific clonal cells and the cardiomyocyte progeny. Following the identification of clonal c-kit-BMCs able and unable to form cardiomyocytes, BMCs were subjected to RNA sequencing.^54^

#### RNA-sequencing

Clonal myogenic c-kit-BMCs and freshly isolated FACS-sorted c-kit-BMCs were utilized in this assay. RNA was isolated using an RNeasy mini kit (Qiagen), and 100 ng of total RNA was converted to complementary DNA (cDNA) and amplified using NuGEN V2 RNA-Seq kit (NuGEN). cDNA was sonicated to an average fragment size of 300 bp and Illumina sequencing adapters were ligated to 500 ng of cDNA using NEBNext mRNA Library Prep Reagent Set for Illumina (New England Biolabs). Sequencing was performed using Illumina’s HiSeq2000 platform using paired in reads at an average length of 100 bp. Trimmed reads were mapped to reference genome (UCSC mm10) with TopHat. Cufflinks was used for transcript assembly and the expression profile was calculated for each sample and transcript/gene as FPKM to identify differentially expressed genes (DEGs). In case of known gene annotation, functional annotation and gene-set enrichment analysis were performed on DEGs using Gene Ontology and KEGG database. In the latter case, similarly and differentially expressed gene networks were identified by applying the modified fisher’s exact text followed by Bonferroni and FDR tests. The statistical analysis of the RNA-sequencing data was performed by Macrogen (Amsterdam, The Netherlands).

### Statistical analysis

Data are presented as mean ± SD. *P < *0.05 was considered significant. For the hemodynamic data the two tailed unpaired Student’s *t*-test or Mann–Whitney Rank Sum Test were applied.

### Data availability

The RNA sequencing source data are available as Supplementary Data Set. The datasets generated during and/or analyzed during the current study are available from the corresponding author on reasonable request.

## Electronic supplementary material

Supplemental Material

Supplementary Figure S1

Supplementary Figure S2

Supplementary Figure S3

Supplementary Figure S4

Supplementary Figure S5

Supplementary Figure S6

Supplementary Figure S7

Supplementary Figure S8

Supplementary Figure S9

Supplementary Figure S10

Supplementary Figure S11

Supplementary Figure S12

Dataset 1 RNA sequencing data

## References

[1] Orlic D (2001). Bone marrow cells regenerate infarcted myocardium. Nature.

[2] Afzal MR (2015). Adult bone marrow cell therapy for ischemic heart disease: evidence and insights from randomized controlled trials. Circ. Res..

[3] Tongers J, Losordo DW, Landmesser U (2011). Stem and progenitor cell-based therapy in ischaemic heart disease: promise, uncertainties, and challenges. Eur. Heart J..

[4] van der Spoel TI (2011). Human relevance of pre-clinical studies in stem cell therapy: systematic review and meta-analysis of large animal models of ischaemic heart disease. Cardiovasc. Res..

[5] Golpanian S, Wolf A, Hatzistergos KE, Hare JM (2016). Rebuilding the damaged heart: mesenchymal stem cells, cell-based therapy, and engineered heart tissue. Physiol. Rev..

[6] Balsam LB (2004). Haematopoietic stem cells adopt mature haematopoietic fates in ischaemic myocardium. Nature..

[7] Su F, Zhang W, Liu J (2015). Membrane estrogen receptor alpha is an important modulator of bone marrow C-Kit+cells mediated cardiac repair after myocardial infarction. Int. J. Clin. Exp. Pathol..

[8] Ding R (2015). Activation of Notch1 signalling promotes multi-lineage differentiation of c-Kit(POS)/NKX2.5(POS) bone marrow stem cells: implication in stem cell translational medicine. Stem Cell Res. Ther..

[9] Schmidt M (2001). A model for the detection of clonality in marked hematopoietic stem cells. Ann. N.Y. Acad. Sci..

[10] Schmidt M (2003). Clonality analysis after retroviral-mediated gene transfer to CD34+ cells from the cord blood of ADA-deficient SCID neonates. Nat. Med.

[11] Mazurier F, Gan OI, McKenzie JL, Doedens M, Dick JE (2004). Lentivector-mediated clonal tracking reveals intrinsic heterogeneity in the human hematopoietic stem cell compartment and culture-induced stem cell impairment. Blood.

[12] Seri B (2006). Composition and organization of the SCZ: a large germinal layer containing neural stem cells in the adult mammalian brain. Cereb. Cortex.

[13] Rompani SB, Cepko CL (2008). Retinal progenitor cells can produce restricted subsets of horizontal cells. Proc. Natl. Acad. Sci. USA.

[14] Weber K, Thomaschewski M, Benten D, Fehse B (2011). RGB marking facilitates multicolor clonal cell tracking. Nat. Med..

[15] Qian Li (2012). In vivo reprogramming of murine cardiac fibroblasts into induced cardiomyocytes. Nature.

[16] Nefzger CM, Alaei S, Knaupp AS, Holmes ML, Polo JM (2014). Cell surface marker mediated purification of iPS cell intermediates from a reprogrammable mouse model. J. Vis. Exp..

[17] Mazurier F, Gan OI, McKenzie JL, Doedens M, Dick JE (2004). Lentivector-mediated clonal tracking reveals intrinsic heterogeneity in the human hematopoietic stem cell compartment and culture-induced stem cell impairment. Blood..

[18] Anzai N (2002). C-kit associated with the transmembrane 4 superfamily proteins constitutes a functionally distinct subunit in human hematopoietic progenitors. Blood.

[19] Abdelli LS, Singla DK (2015). A CD63(+ve)/c-kit(+ve) stem cell population isolated from the mouse heart. Mol. Cell. Biochem.

[20] Khurana S (2013). A novel role of BMP4 in adult hematopoietic stem and progenitor cell homing via Smad independent regulation of integrin-α4 expression. Blood..

[21] Hatzistergos KE (2015). cKit+ cardiac progenitors of neural crest origin. Proc. Natl. Acad. Sci. USA.

[22] Mehta G (2015). MITF interacts with the SWI/SNF subunit, BRG1, to promote GATA4 expression in cardiac hypertrophy. J. Mol. Cell. Cardiol..

[23] Samse K, Hariharan N, Sussman MA (2016). Personalizing cardiac regenerative therapy: at the heart of Pim1 kinase. Pharmacol. Res..

[24] Li G (2009). Comparative proteomic analysis of mesenchymal stem cells derived from human bone marrow, umbilical cord, and placenta: implication in the migration. Proteomics..

[25] Giordano S (2002). The semaphorin 4D receptor controls invasive growth by coupling with Met. Nat. Cell. Biol..

[26] Delaloy C (2010). MicroRNA-9 coordinates proliferation and migration of human embryonic stem cell-derived neural progenitors. Cell Stem Cell.

[27] Cohen ED, Tian Y, Morrisey EE (2008). Wnt signaling: an essential regulator of cardiovascular differentiation, morphogenesis and progenitor self-renewal. Development..

[28] Li TS (2005). Regeneration of infarcted myocardium by intramyocardial implantation of ex vivo transforming growth factor-beta-preprogrammed bone marrow stem cells. Circulation.

[29] Gude N (2015). Notch activation enhances lineage commitment and protective signaling in cardiac progenitor cells. Basic Res. Cardiol..

[30] Georgakilas AG, Martin OA, Bonner WM (2017). p21: A two-faced genome guardian. Trends Mol. Med..

[31] Rota M (2007). Bone marrow cells adopt the cardiomyogenic fate in vivo. Proc. Natl. Acad. Sci. USA.

[32] Etzrodt M, Endele M, Schroeder T (2014). Quantitative single-cell approaches to stem cell research. Cell Stem Cell.

[33] Wagers AJ, Sherwood RI, Christensen JL, Weissman IL (2002). Little evidence for developmental plasticity of adult hematopoietic stem cells. Science.

[34] Fazel S (2006). Cardioprotective c-kit+ cells are from the bone marrow and regulate the myocardial balance of angiogenic cytokines. J. Clin. Invest..

[35] Xayamardan M (2009). c-Kit function is necessary for in vitro myogenic differentiation of bone marrow hematopoietic cells. Stem Cells.

[36] Loffredo FS, Steinhauser ML, Gannon J, Lee RT (2011). Bone marrow-derived cell therapy stimulates endogenous cardiomyocyte progenitors and promotes cardiac repair. Cell Stem Cell.

[37] Wu JM, Turcotte R, Alt C, Runnels JM, Tsao H, Linb CH (2016). Defining clonal color in fluorescent multi-clonal tracking. Sci. Rep..

[38] Wu JM (2015). Circulating cells contribute to cardiomyocyte regeneration after injury. Circ. Res..

[39] Nygren JM (2008). Myeloid and lymphoid contribution to non-haematopoietic lineages through irradiation-induced heterotypic cell fusion. Nat. Cell Biol..

[40] Fukata M (2013). Contribution of bone marrow-derived hematopoietic stem/progenitor cells to the generation of donor-marker^+^ cardiomyocytes in vivo. PLoS ONE.

[41] Nygren JM (2004). Bone marrow-derived hematopoietic cells generate cardiomyocytes at a low frequency through cell fusion, but not transdifferentiation. Nat. Med..

[42] Quijada P, Sussman MA (2015). Circulating around the tissue: hematopoietic cell-based fusion versus transdifferentiation. Circ. Res..

[43] Kawamoto A (2006). CD34-positive cells exhibit increased potency and safety for therapeutic neovascularization after myocardial infarction compared with total mononuclear cells. Circulation.

[44] Dey D (2013). Dissecting the molecular relationship among various cardiogenic progenitor cells. Circ. Res..

[45] Tang XL (2016). Long-term outcome of administration of c-kit^pos^ cardiac progenitor cells after acute myocardial infarction: transplanted cells do not become cardiomyocytes, but structural and functional improvement and proliferation of endogenous cells persist for at least one year. Circ. Res..

[46] Liu X (2015). Rescue of neonatal cardiac dysfunction in mice by administration of cardiac progenitor cells in utero. Nat. Commun..

[47] Yonemura Y, Ku H, Lyman SD, Ogawa M (1997). In vitro expansion of hematopoietic progenitors and maintenance of stem cells: comparison between FLT3/FLK-2 ligand and KIT ligand. Blood.

[48] Hosoda T (2009). Clonality of mouse and human cardiomyogenesis in vivo. Proc. Natl. Acad. Sci. USA..

[49] Hui, E. K-W., Wang, P-C. & Lo, S. J. PCR-based strategies to clone unknown DNA regions from known foreign integrants: an overview. In *PCR Cloning Protocols*, Chen B-Y, Janes HW (eds) p. 249–274 (Humana Press, New York, 2000). 10.1385/1-59259-177-9:249 12494657

[50] Weber K, Thomaschewski M, Benten D, Fehse B (2012). RGB marking with lentiviral vectors for multicolor clonal cell tracking. Nat. Protoc..

[51] Cornils K (2014). Multiplexing clonality: combining RGB marking and genetic barcoding. Nucl. Acid. Res..

[52] Signore S (2015). Late Na(+) current and protracted electrical recovery are critical determinants of the aging myopathy. Nat. Commun..

[53] Meo M (2016). Reduction in Kv current enhances the temporal dispersion of the action potential in diabetic myocytes: insights from a novel repolarization algorithm. J. Am. Heart Assoc..

[54] Trapnell C (2010). Transcript assembly and quantification by RNA-Seq reveals unannotated transcripts and isoform switching during cell differentiation. Nat. Biotech..

