# Supplementary material for: Single-cell analysis of the fate of c-kit-positive bone marrow cells
Source: NPJ Regen Med. 2017 Oct 16;2:27. doi: 10.1038/s41536-017-0032-1 (PMC5678002; doi:10.1038/s41536-017-0032-1)
Supplement: Supplementary file 1 — Supplemental Material [file 41536_2017_32_MOESM1_ESM.pdf]

Legends to Supplementary Figures (Please, for details in the illustrations view the images on the computer-screen)

Supplementary Figure S1. Sequence analysis of PCR products. Examples of DNA sequences comprising the viral (green line) and mouse (black line) genome. The magenta line corresponds to the Taq I digestion site.

Supplementary Figure S2. Lentiviral integration sites in c-kit-BMCs and their progeny in vivo. **a** Chromosome number, length of key DNA sequences and the closest gene to the viral integration site are listed. **b** Sites of integration (IS) of the viral genome in the myocardium of 7 mice: myocytes (red dots), endothelial cells (ECs; blue dots), fibroblasts (yellow dots) and c-kit- BMCs (green dots). No sites of integration were detected in animal number 6.

Supplementary Figure S3. c-kit expression in BMCs. Dot plots show that a subset of BMCs are positive for c-kit (lower panels). Please note the variability in brightness of APC-c-kit, indicative of a non-uniform expression level of the receptor tyrosine kinase within this cell population. Isotype controls are illustrated in the upper panels.

Supplementary Figure S4. Functional classification of DEGs in myogenic clonal c-kit-BMCs vs. c-kit-BMCs. Enrichment map (KEGG database) of annotated gene sets. The color gradient shows the statistical significance based on *P* value.

Supplementary Figure S5. Hematopoietic lineage pathway map (KEGG database). Red star: statistically significant different pathway modules. Green colored boxes: modules that are not mapped. White colored boxes: modules that are not relevant to the species. The color gradient on the right corner indicates the difference in fold-change of gene expression in myogenic c-kit-BMCs vs. whole bone marrow.

Supplementary Figure S6. Wnt signaling pathway map (KEGG database). See Figure S4 for description.

Supplementary Figure S7. HIF-1 signaling pathway map (KEGG database). See Figure S4 for description.

Supplementary Figure S8. PI3K-AKT signaling pathway map (KEGG database). See Figure S4 for description.

Supplementary Figure S9. Notch signaling pathway map (KEGG database). See Figure S4 for description.

Supplementary Figure S10. c-kit-BMCs regenerate the infarcted myocardium. The images in panels a-c were collected 4 to 7 days after infarction and cell delivery. **a** Below a thin layer of spared endomyocardium (EM), the infarcted region is replaced by a large number of small fluorescently labeled cells. A cocktail of anti-mCherry and anti-CFP was employed to identify the progeny of c-kit-BMCs (green). In the EM, cardiomyocytes are positive for troponin I (TnI; red). Scale bar = 100 μm. **b, c** Small newly-formed cells (green), at times positive for GATA4 (b: red dots in nuclei; arrows) and Nkx2.5 (c: red dots in nuclei; arrowheads), are present between spared cardiomyocytes positive for α-sarcomeric actin (α-SA, gray-white). The areas included in the squares are shown at higher magnification in the insets. In panel b, the inset illustrates GATA4 labeling (red dots) on the left and α-SA labeling (gray-white) on the right. In panel c, the inset illustrates Nkx2.5 labeling (red dots) on the left and α-SA labeling (gray-white) on the right. A cocktail of anti-mCherry, anti-YFP and anti-CFP was employed to identify the progeny of c-kit- BMCs (green). Scale bars = 20 μm.

Supplementary Figure S11. Differentiation of c-kit-BMCs into coronary vessels. **a** Small vessels defined by an endothelial lining labeled by YFP (green) and CD31 (red). Scale bar = 20 μm. Two of these vessels (yellow arrows) are illustrated at higher magnification in the insets (right panels, scale bar = 10 μm.) where the individual channels for YFP and CD31 are shown. White arrowheads point to cells positive for both YFP and CD31. **b** Coronary

arterioles (yellow arrows) were stained with a cocktail of mCherry, YFP and CFP (green). ECs are positive for CD31 (red) and smooth muscle cells for α- smooth muscle actin (α-SMA, blue). Scale bar = 20 µm. Two of these arterioles (yellow arrows) are illustrated at higher magnification in the insets (right panels, scale bar = 10 µm.) where the individual channels for mCherry-YFP- CFP (green), CD31 (red) and α-SMA (blue) are shown.

Supplementary Figure S12. c-kit-BMCs expand clonally and regenerate the infarcted myocardium. Images in panels a-c were collected 14 to 21 days after infarction and cell delivery. **a** At 21 days, the infarcted myocardium is almost completely replaced by newly-formed small cells (green). Scale bar = 50 µm. As examples, the cells pointed by the three yellow arrowheads numbered 1-3 are illustrated at higher magnification in the insets (right, small panels, scale bar = 5 µm) where the co-localization of GATA4 (red) and α-SA (white) is apparent. EM: endomyocardium. **b, c** Additional examples in which mCherry, YFP and CFP positive cells (green; left panels) express GATA4 (red) and α-SA (white; right panels). A cocktail of anti-mCherry, anti-YFP, and anti-CFP was employed to identify the progeny of c-kit-BMCs (green). Panel b, scale bar = 20 µm; Panel c, scale bar = 10 µm.

Legend to Supplementary Data Set

Supplementary Data Set S1. RNA sequencing raw data.
