# Supplementary figures and images for: Single-cell analysis of the fate of c-kit-positive bone marrow cells
Source: NPJ Regen Med. 2017 Oct 16;2:27. doi: 10.1038/s41536-017-0032-1 (PMC5678002; doi:10.1038/s41536-017-0032-1)

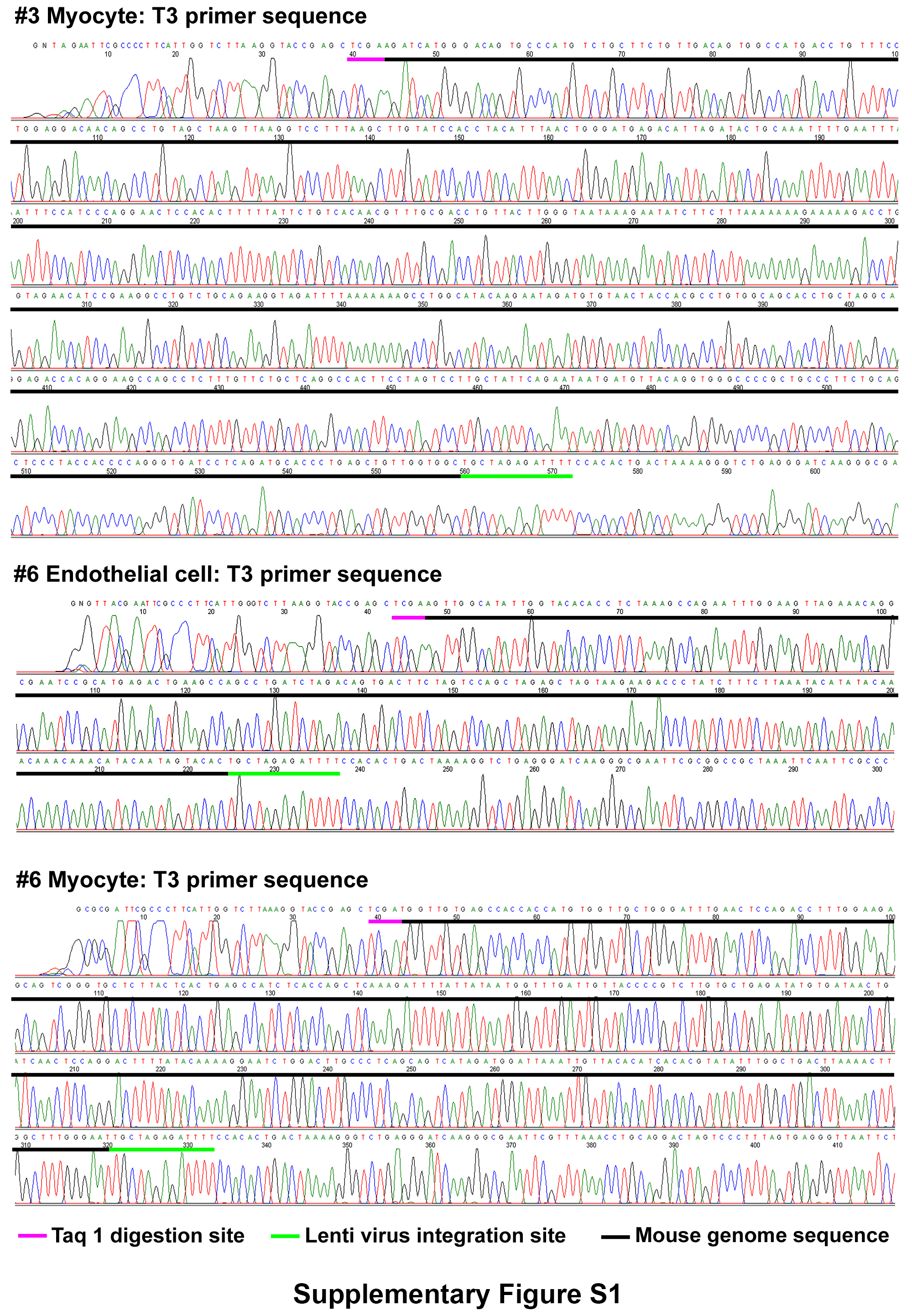

Supplement: Supplementary file 2 — Supplementary Figure S1 [file 41536_2017_32_MOESM2_ESM.jpg]

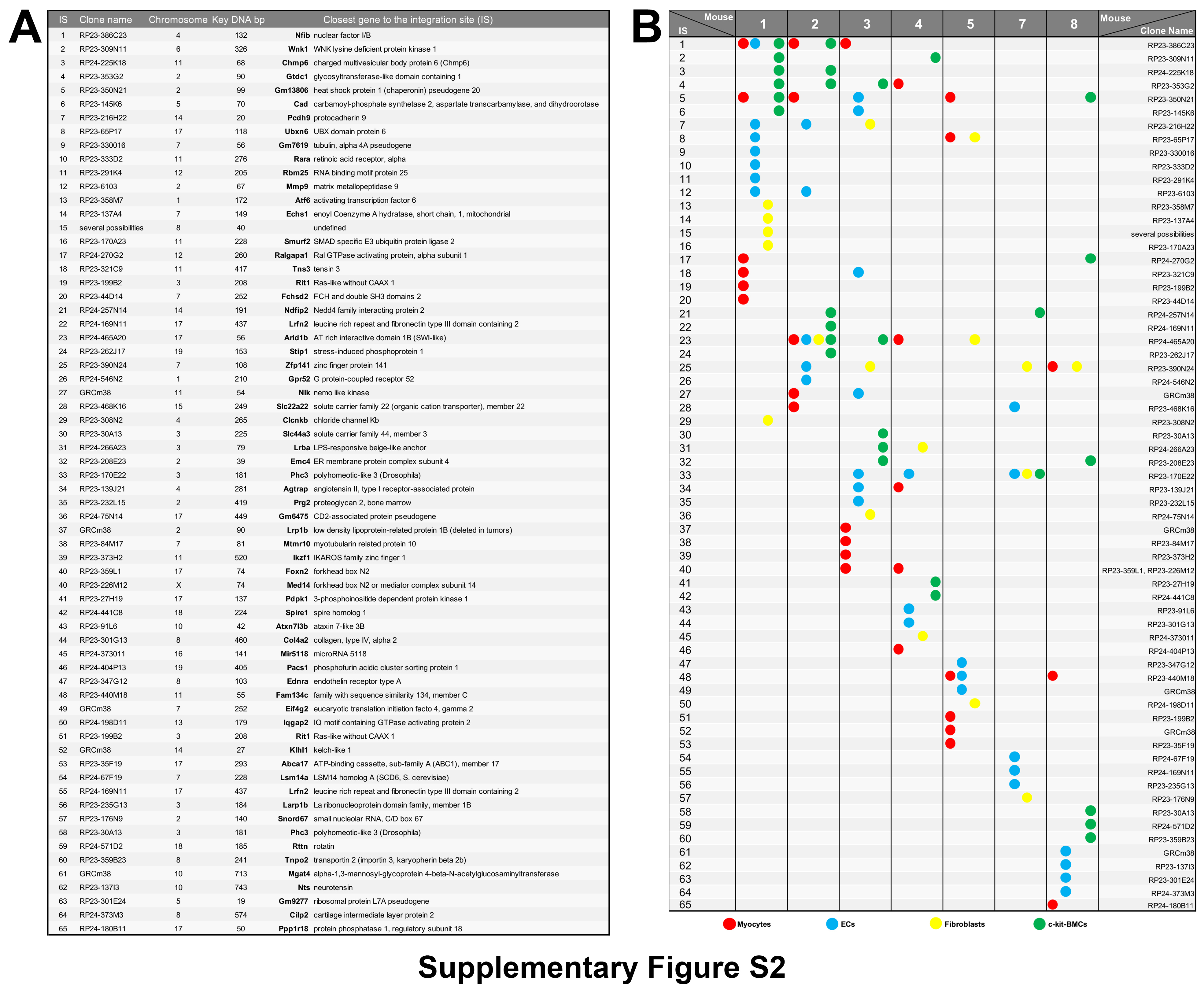

Supplement: Supplementary file 3 — Supplementary Figure S2 [file 41536_2017_32_MOESM3_ESM.jpg]

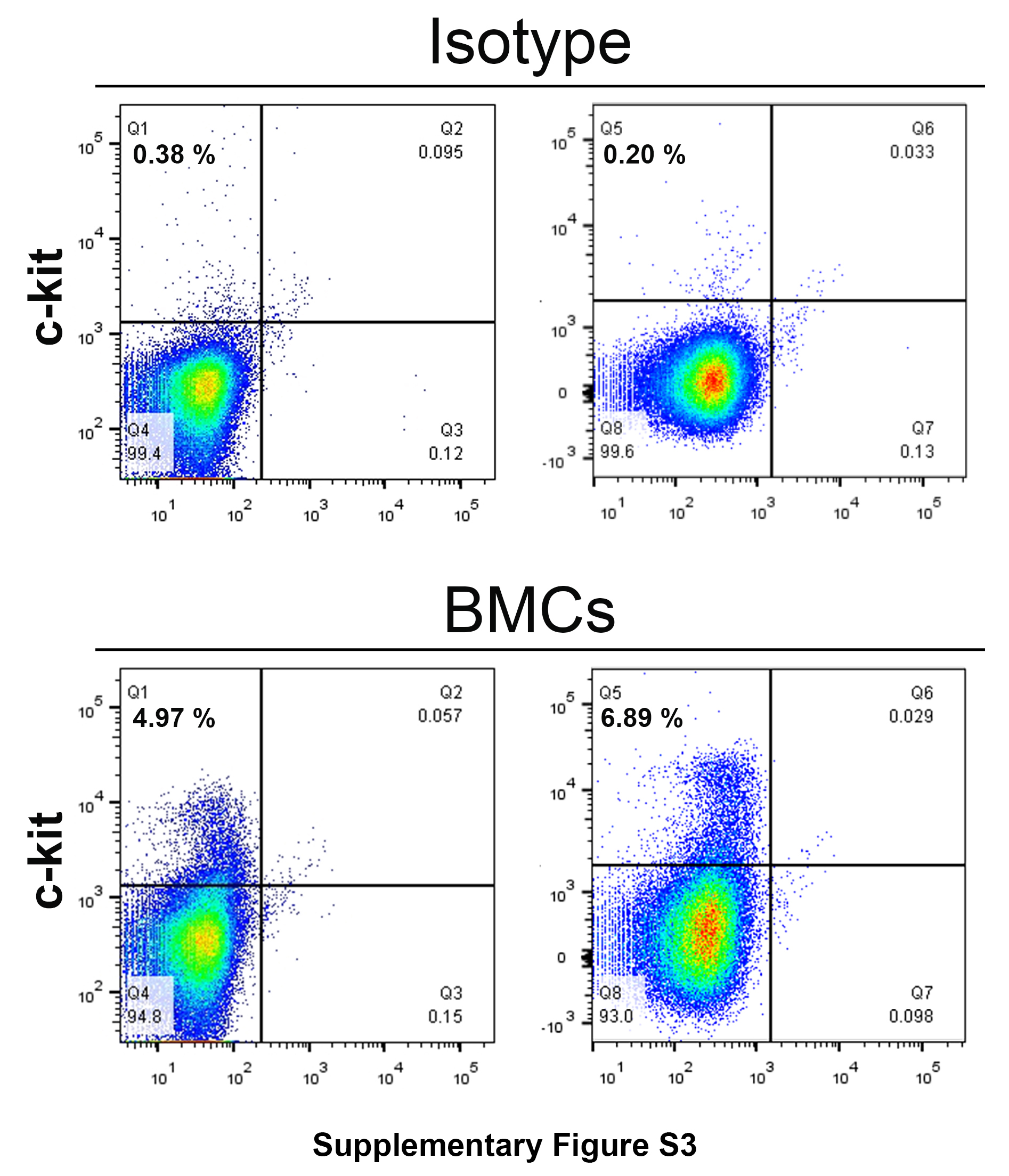

Supplement: Supplementary file 4 — Supplementary Figure S3 [file 41536_2017_32_MOESM4_ESM.jpg]

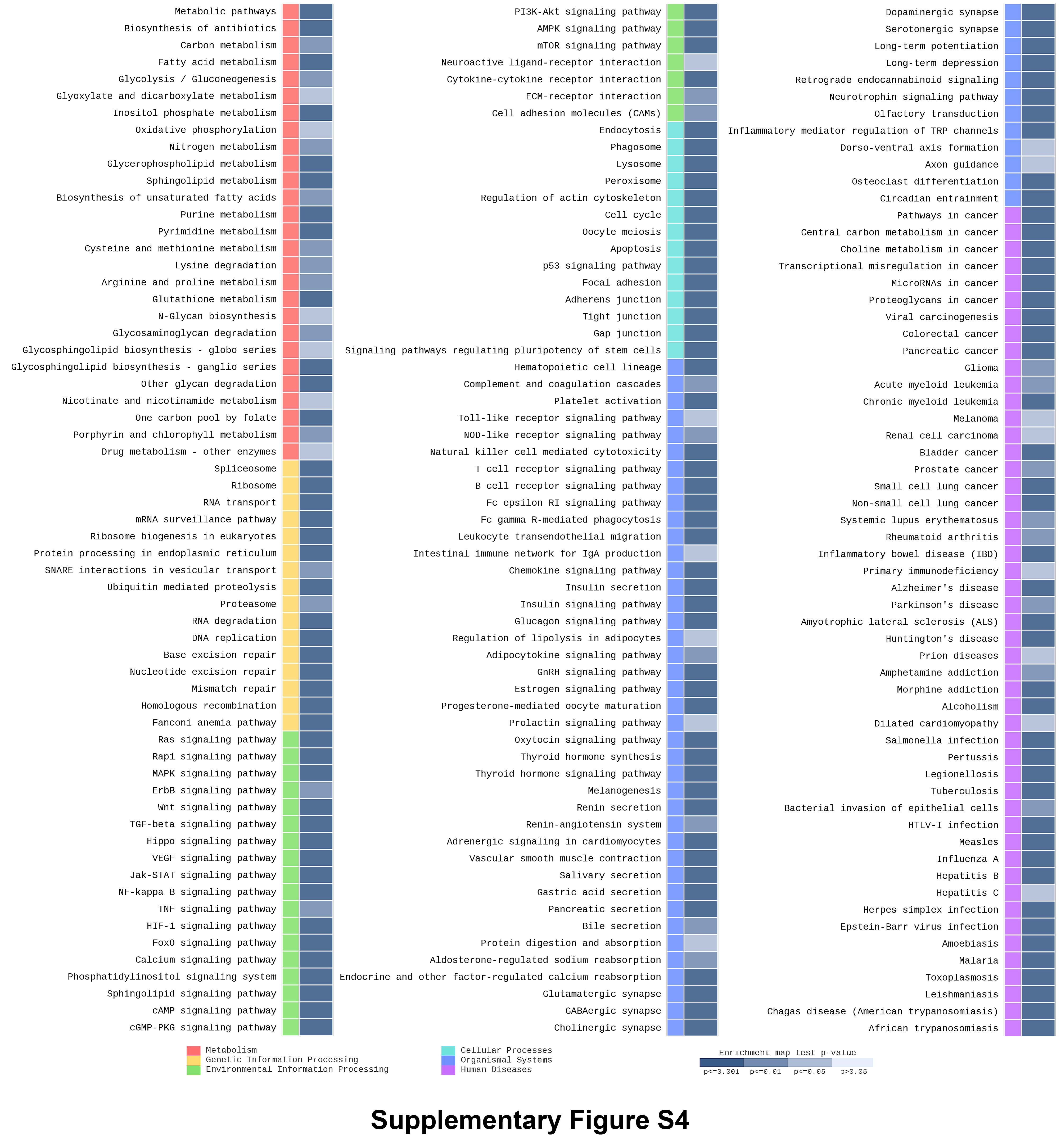

Supplement: Supplementary file 5 — Supplementary Figure S4 [file 41536_2017_32_MOESM5_ESM.jpg]

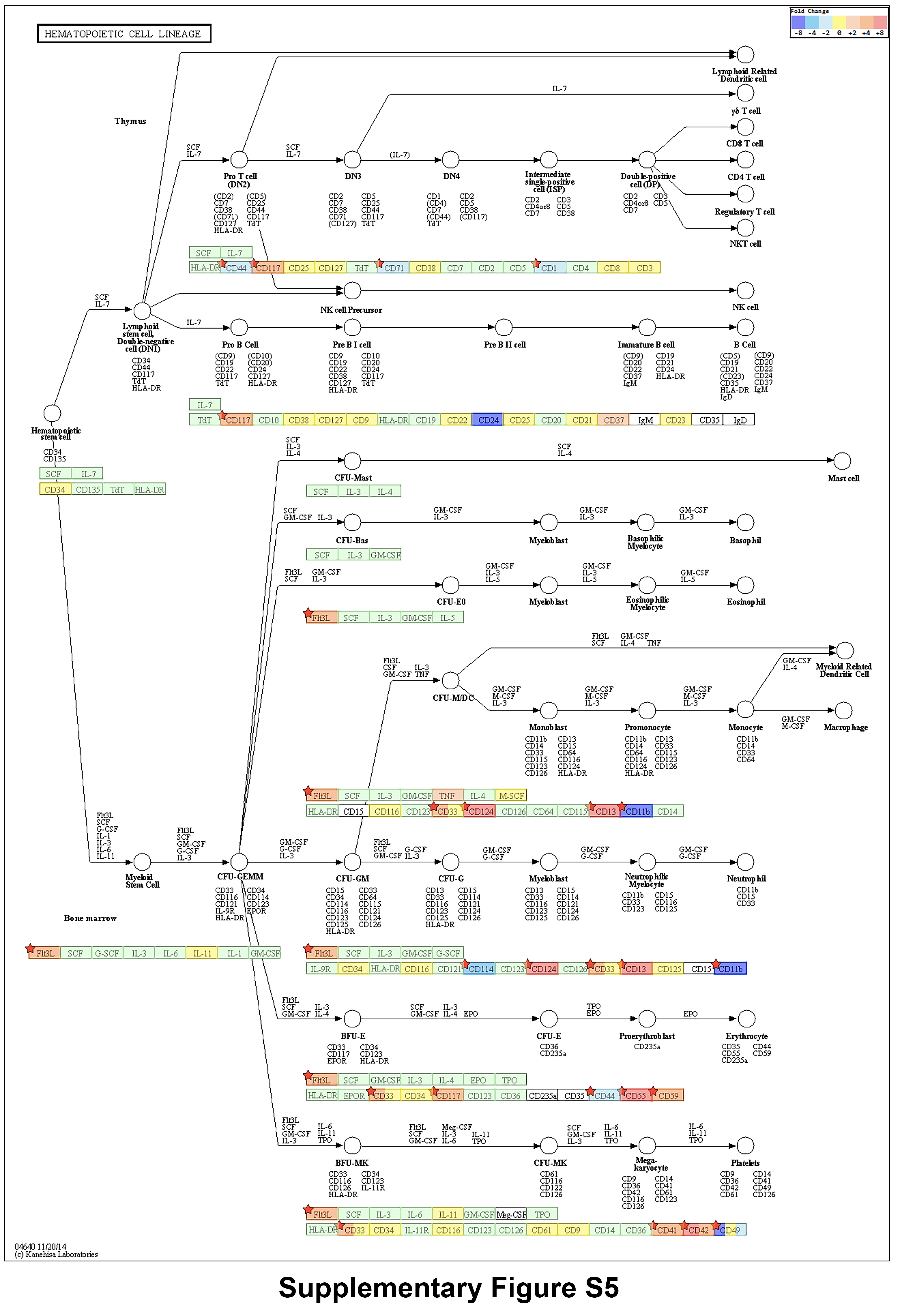

Supplement: Supplementary file 6 — Supplementary Figure S5 [file 41536_2017_32_MOESM6_ESM.jpg]

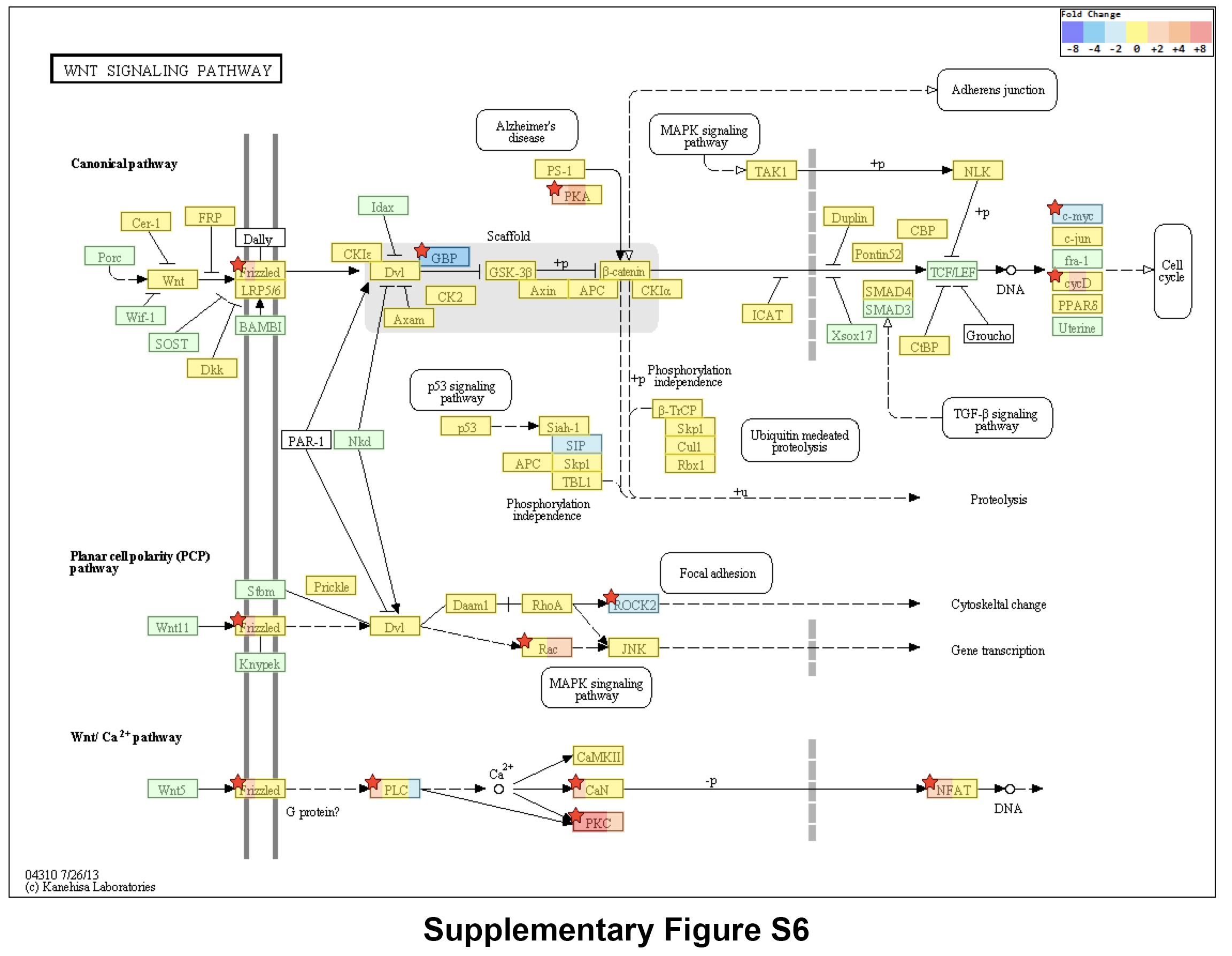

Supplement: Supplementary file 7 — Supplementary Figure S6 [file 41536_2017_32_MOESM7_ESM.jpg]

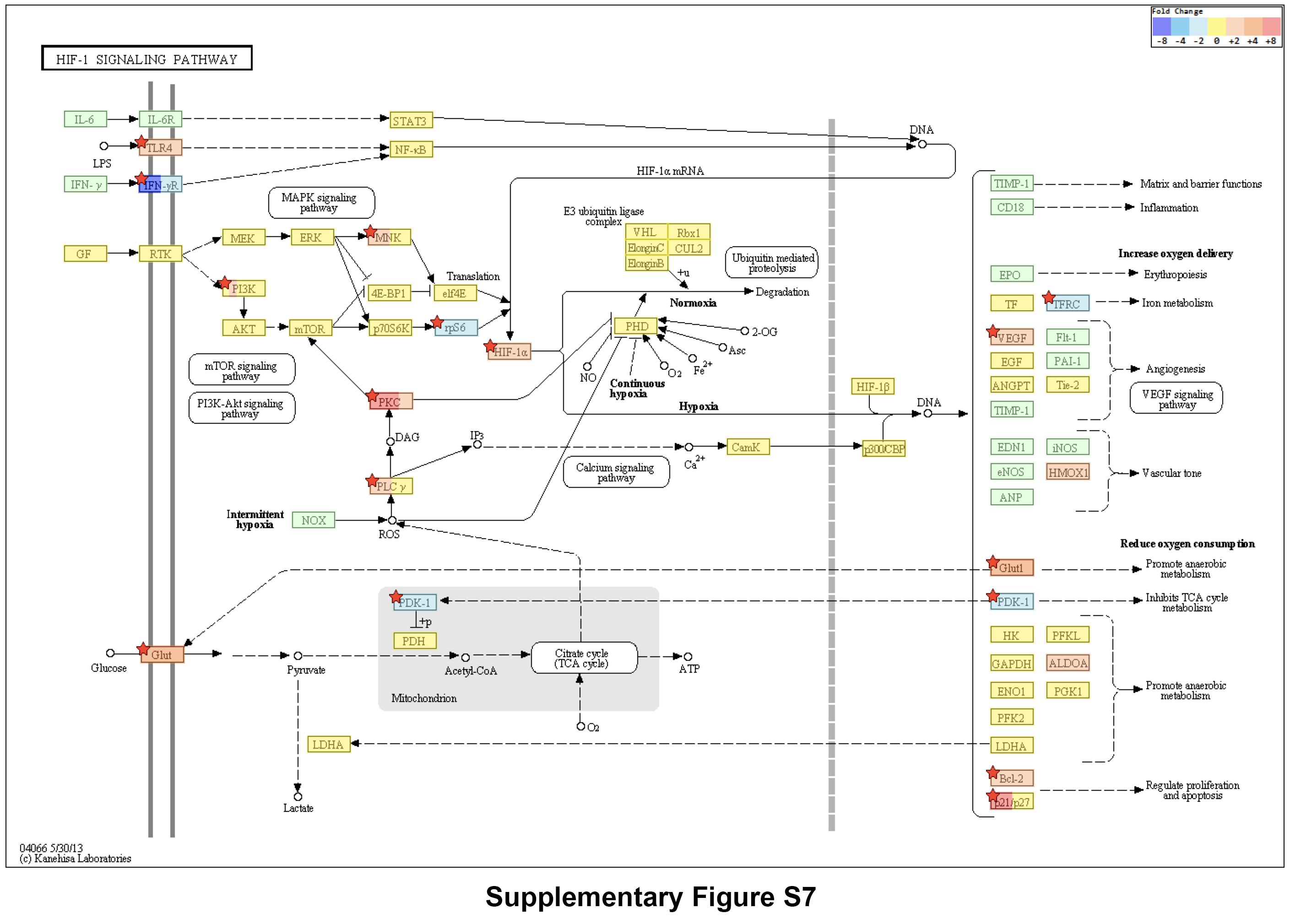

Supplement: Supplementary file 8 — Supplementary Figure S7 [file 41536_2017_32_MOESM8_ESM.jpg]

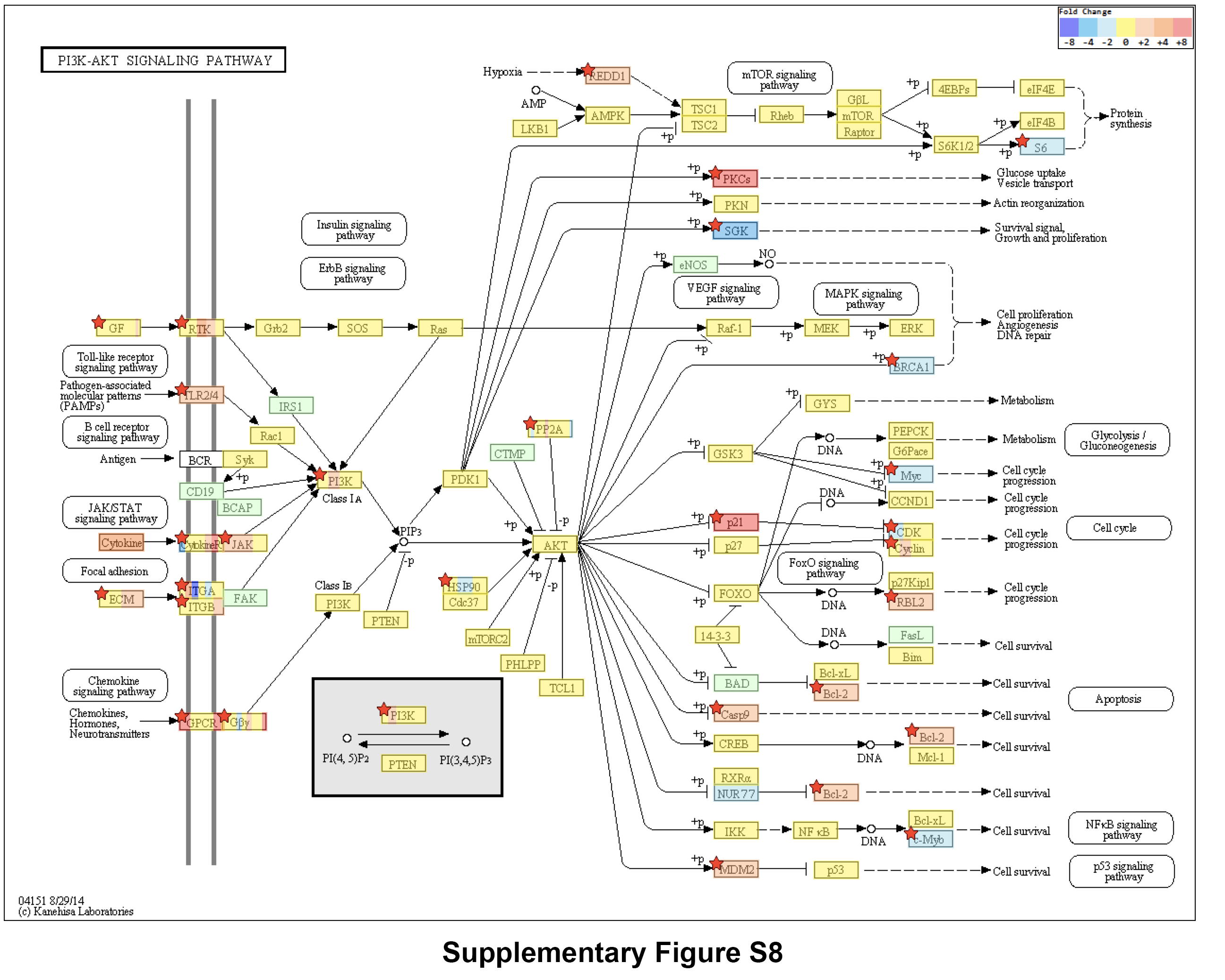

Supplement: Supplementary file 9 — Supplementary Figure S8 [file 41536_2017_32_MOESM9_ESM.jpg]

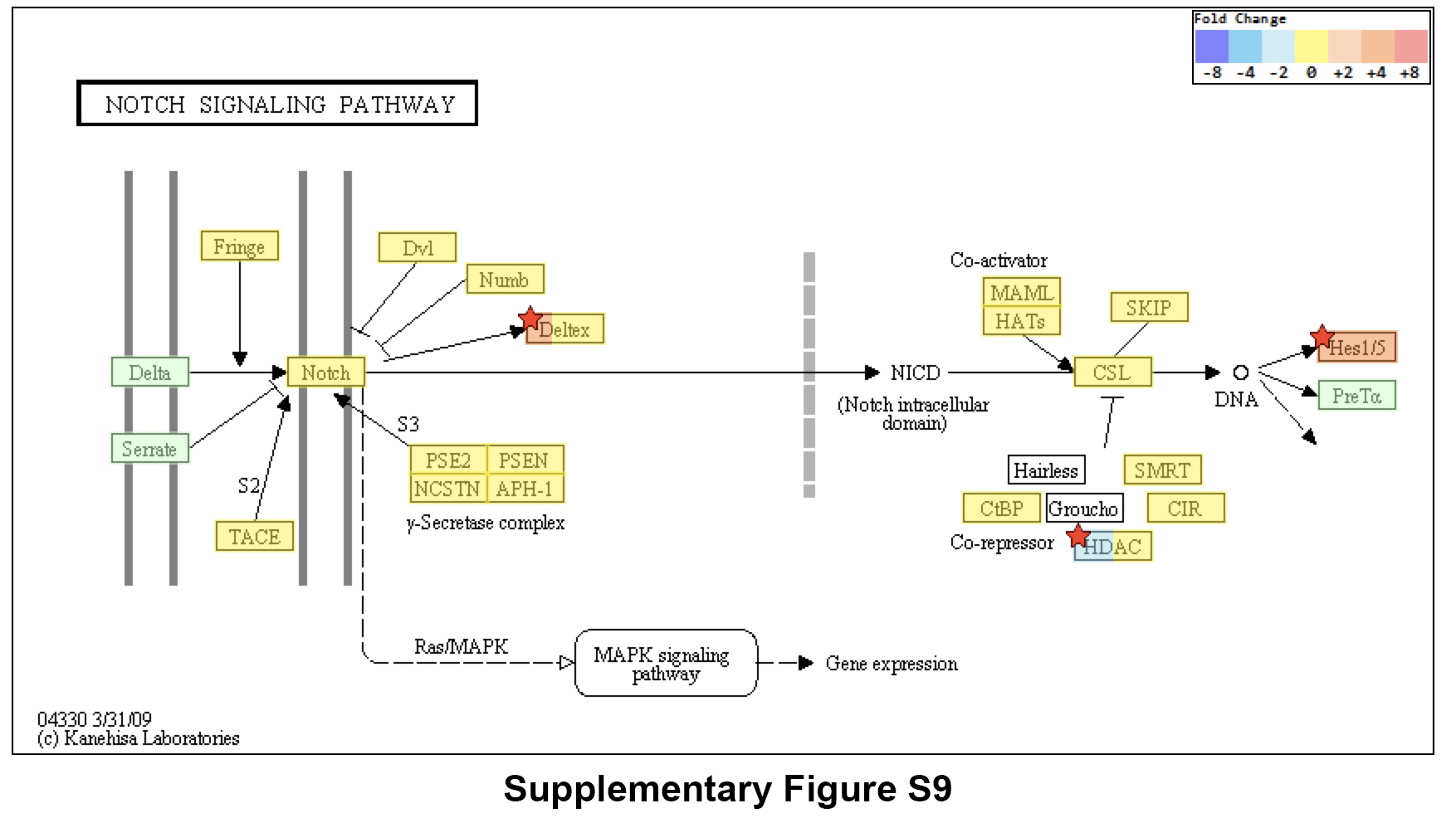

Supplement: Supplementary file 10 — Supplementary Figure S9 [file 41536_2017_32_MOESM10_ESM.jpg]

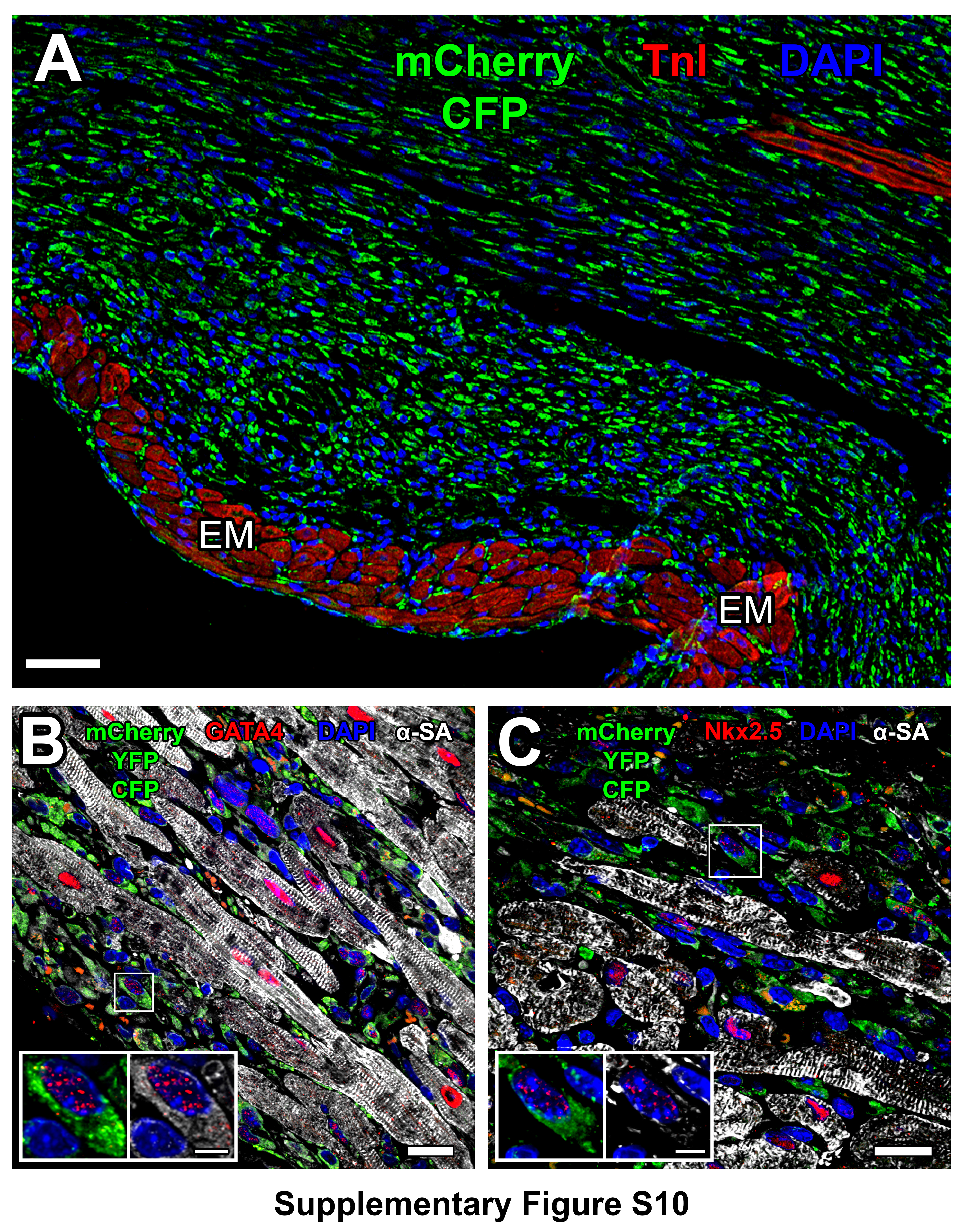

Supplement: Supplementary file 11 — Supplementary Figure S10 [file 41536_2017_32_MOESM11_ESM.jpg]
